# Funding rules that promote equity in climate adaptation outcomes

**DOI:** 10.1073/pnas.2418711121

**Published:** 2025-01-07

**Authors:** Adam B. Pollack, Sara Santamaria-Aguilar, Pravin Maduwantha, Casey Helgeson, Thomas Wahl, Klaus Keller

**Affiliations:** ^a^Thayer School of Engineering, Dartmouth College, Hanover, NH 03755; ^b^Department of Civil, Environmental and Construction Engineering, University of Central Florida, Orlando, FL 32816; ^c^National Center for Integrated Coastal Research, University of Central Florida, Orlando, FL 32816; ^d^Earth and Environmental Systems Institute, Penn State University, State College, PA 16801; ^e^Department of Philosophy, Penn State University, State College, PA 16801

**Keywords:** equity, justice40, flood-risk management, climate adaptation

## Abstract

Many climate policies adopt improving equity as a key objective. A key challenge is that policies often conceive of equity in terms of individuals but introduce strategies that focus on spatially coarse administrative areas. For example, the Justice40 Initiative in the United States requires 518 diverse federal programs to prioritize funds for “disadvantaged” census tracts. This strategy is largely untested and contrasts with the federal government’s definition of equity as the “consistent and systematic fair, just and impartial treatment of all individuals (Executive Office of the President, Federal Register, 2021).” How well does the Justice40 approach improve equity in climate adaptation outcomes across *individuals*? We analyze this question using a case study of a municipality that faces repetitive flooding and struggles to effectively manage these risks due to limited resources and public investment. We find that the way the Federal Emergency Management Agency implements the Justice40 Initiative can be an obstacle to promoting equity in household flood-risk outcomes. For example, in this case study, ensuring the majority of benefits accrue in “Justice40 Communities” does not reduce risk for the most burdened households, does not reduce risk-burden inequality, and produces net costs. In contrast, we design simple funding rules based on household risk burden that cost-effectively target the most burdened households, reduce risk-burden inequality, and accrue large net benefits. Our findings suggest that “disadvantaged community” indicators defined at coarse spatial scales face the risk of poorly capturing many climate risks and can be ineffective for meeting equity promises about climate-related investments.

## Significance

Public management of climate risks often aims to promote equity. One key approach to this end is through adaptation funding rules. In the United States, the Justice40 Initiative, introduced in 2021, transformed the way federal agencies prioritize grant applications for funding related to climate investments. We analyze whether the Justice40 Initiative can improve on equity in household outcomes in a flood elevation case study in a resource-stressed municipality. In our case study, prioritizing funds for elevation in “Justice40 Communities” leads to less equitable outcomes across households compared to all other considered funding rules. We design and test simple funding rules based on household risk burden that can improve on Justice40 in terms of both equity *and* economic objectives.

Promoting equity in climate adaptation outcomes is an emerging policy priority around the world (1–8). In the United States, this goal was formally stated in a 2021 Executive Order on “Tackling the Climate Crisis at Home and Abroad” that outlines policies for “securing environmental justice and spurring economic opportunity (7).” The Order introduced the Justice40 Initiative to achieve this goal, which requires that 40 percent of the overall benefits from federal investments flow to “disadvantaged communities.” The White House instructs federal agencies to use the Climate and Economic Justice Screening Tool (CEJST) definition of “disadvantaged communities” for all 518 programs covered by the Initiative (9, 10).

Several agencies implement the Justice40 Initiative by prioritizing grant applications in which a majority of benefits accrue in CEJST-identified “disadvantaged communities” (7, 9–12). This new prioritization approach allocates billions of dollars in annual federal expenditures (7, 9, 11–15). However, this approach is largely untested for whether it promotes equity across individuals, the scale at which the federal government defines equity (1, 16). This gap is especially relevant for Justice40-covered programs that fund household-level interventions, such as those administered by the Federal Emergency management Agency (FEMA) (7, 9–12). Here, we address this gap by assessing two fundamental and highly policy-relevant research questions.

**Fig. 1.**
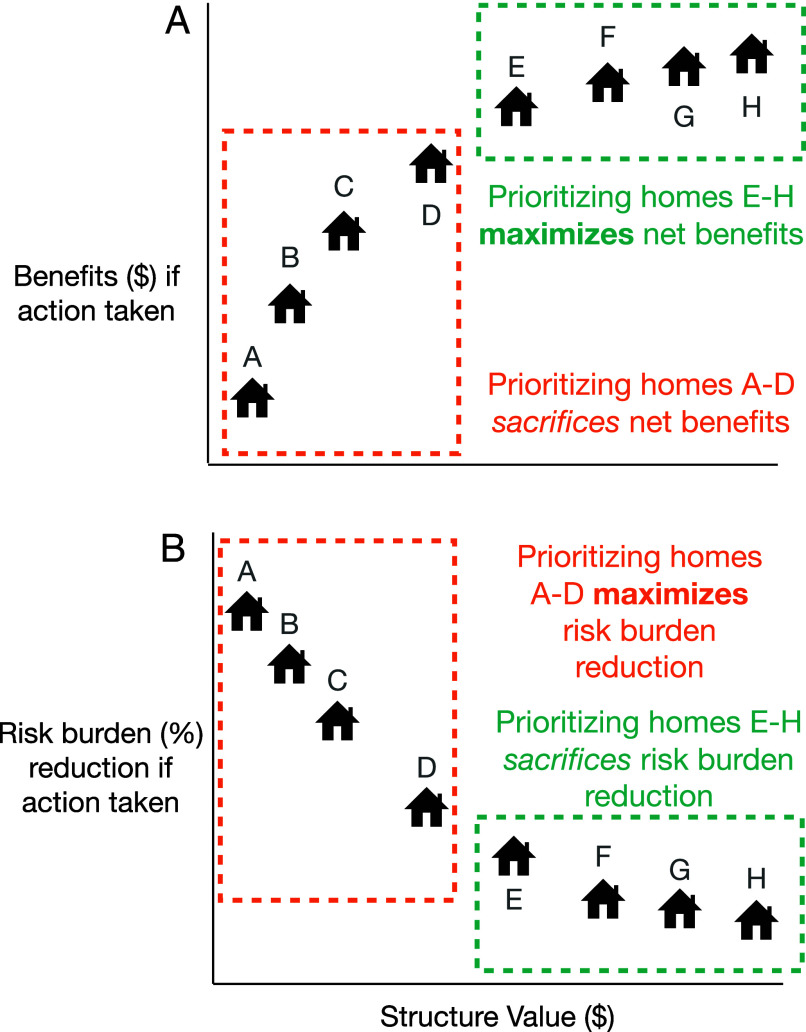
Illustration of how different funding rules can induce tradeoffs between equity and economic objectives. In both panels, households are ordered in terms of their structure value on the x-axis. In Panel *A*, the economic metric (y-axis) measures the monetary benefits households receive if a risk intervention action is taken. In Panel *B*, the equity metric (y-axis) measures the reduction in risk burden households experience if the same action is taken. Here, risk burden is defined as benefits from an action as a percentage of structure value. Under a limited budget, a decision-maker must choose a subset of households for an intervention. If the decision-maker prioritizes the economic objective of “maximize net benefits,” they may choose homes with higher structure value if action is taken (Panel *A*) than if they prioritize the equity objective of “minimize risk burden” (Panel *B*).

First, can climate-adaptation grant funding rules that require the majority of benefits flow to “disadvantaged communities” promote equity in household outcomes? We focus on this scale mismatch because community-level prioritization may be a poor approach to promote equity in household outcomes. In particular, community-level prioritization (e.g., census tract or block group) is subject to aggregation bias, posing a risk that reliance on coarse indicators may overlook households facing fine-scaled economic and environmental burdens (17–28) (e.g., if houses *A*–*D* in Fig. 1 are not in a “disadvantaged community”). On the other hand, even if “disadvantaged communities” capture the most-burdened households, if these households do not accrue large enough benefits, an application that includes these households may not meet the “majority of benefits” constraint.

There is scant evidence on whether the community-level funding rules introduced to abide by the Justice40 Initiative can promote equity across households. Reporting about Justice40-covered programs occasionally tracks expenditures across “disadvantaged communities” but does not report on equity across households (7, 29, 30). Based on recent reviews and analyses focused on equity in climate-risk management settings, we are not aware of prior research that addresses this question (6, 8, 31).

Second, do grant funding rules that improve on equity introduce tradeoffs with economic objectives? This is important because the Executive Order (7) highlights environmental justice and economic goals, but research suggests these objectives may be in conflict. For example, several qualitative studies suggest that prioritizing funds based on net benefit, the main economic objective for US federal investment (32), may direct funds away from the most-burdened populations (Fig. 1) (33–36). We are not aware of prior research that quantitatively investigates how well funding rules navigate tradeoffs between equity and economic objectives in allocating limited adaptation funding for property-level interventions.

Previous quantitative studies produce important insights about inequities in public recovery and grant programs. However, they typically do not measure equity at household scales, do not measure the distribution of benefits that the Justice40 Initiative calls for, and do not evaluate the design of funding rules to manage potential tradeoffs (37–48). Notably, one study estimates that prioritizing grant funding in low- and moderate-income block groups can cost-effectively accrue economic benefits, suggesting equity and efficiency synergies (42). However, this study does not evaluate which households benefitted or whether alternative funding rules perform better on equity and economic objectives.

We address knowledge gaps about scale mismatch and potential equity-efficiency tradeoffs in a case study on how well different funding rules promote equity and economic objectives from FEMA household elevation grants in an environmentally burdened and resource-stressed municipality. The decision on how to allocate FEMA household elevation grants is instructive and relevant for our research questions. First, there is a substantial body of literature on aspects of FEMA programs that can lead to inequitable outcomes (34, 37–40, 48–50). Second, FEMA now funds property-level interventions for flood-risk reduction under two Justice40-covered programs (11, 12). Third, a flood-risk focus allows us to build on recent advances in developing equity measurements for flood-risk management (8, 42–44, 51). Finally, FEMA elevation grants focus on flood-risk reduction, which corresponds to burden categories from CEJST (11, 12, 52). Because these elevation grants target specific households, this connection allows us to define household-level equity metrics that are relevant to FEMA’s Justice40-covered programs.

In this case study, we define equity objectives based on a measure of flood-risk burden. This metric, sometimes called relative risk, avoids the influence of wealth on damage that can skew risk and benefit estimates (33, 34, 36, 45, 53). To avoid this skew, which previous research suggests contributes to inequitable disaster aid outcomes (37–39, 48, 54), we define flood-risk burden as the expected annual flood damage as a fraction of a house’s value. This normalization can help avoid inequitable situations, such as grants prioritizing a $500,000 house with 10% damage over a $49,999 house with 100% damage.

Our flood-risk burden metric captures the relative economic burden on a household driven by its exposure to flooding, a type of environmental burden. As such, this metric is very similar to how CEJST identifies “disadvantaged communities” in its climate change burden category (52), except our metric is at the household instead of census tract scale. We note that some conceptions of equity consider the same level of our flood-risk burden metric as a larger burden on lower-income than higher-income households, a notion that distributional weights can handle (47, 55, 56). However, we do not define a weighted metric here because we aim to be consistent with CEJST.

Because equity is a pluralistic concept, we define multiple equity objectives. The first equity objective is to minimize the highest remaining risk burden across households at risk. This reflects the familiar distributive principle that investments should prioritize those who are most burdened (8, 33, 34, 53). Our interpretation is that investments that do not prioritize the households with the highest risk burden are inconsistent with this notion of equity. The second equity objective is to minimize inequality in the share of residual risk burden across households with different structure values. This distributive principle addresses potential concerns about the redistribution of flood-risk burden after a policy is enacted (42, 43, 57). Our interpretation is that investments are more equitable if they lead to greater reductions in risk-burden inequality (51).

We evaluate community-level funding rules, such as those introduced to abide by the Justice40 Initiative, for how well they meet these two equity objectives and how much they accrue overall net benefit under typical FEMA elevation project budgets. We choose the net benefit objective because it is the main economic objective in benefit–cost analysis for public projects and is ubiquitous in research (32, 42, 43, 47, 58–60). In addition to community-level funding rules used in practice, we also design and evaluate simple funding rules based on household-level flood-risk burden. We focus on simple alternative rules, as opposed to finding optimal polices (61, 62), to emphasize scalability and transferability to practical settings. Notably, these household rules are as interpretable as current community-level rules (Table 1) but less complex to computationally implement (*SI Appendix*).

**Table 1.** Funding rules considered in this case study for prioritizing a limited adaptation budget

Funding rule	Spatial scale	Description
Net benefit	Household	Allocate budget to households based on highest to lowest net benefit from elevating a structure to its economically optimal elevation height.
Risk burden reduction	Household	Allocate budget to households based on highest to lowest risk burden reduction from elevating a structure to its economically optimal elevation height.
Initial risk burden	Household	Allocate budget to households based on highest to lowest initial risk burden.
Low-mod income	Census block group	Allocate budget to households based on highest to lowest net benefit from elevating a structure to its economically optimal elevation height. The majority of benefits must accrue across households in Federal Housing Administration (FHA) low- and moderate-income census block groups.
CDC socially vulnerable	Census tract	Allocate budget to households based on highest to lowest net benefit from elevating a structure to its economically optimal elevation height. The majority of benefits must accrue across households in Center for Disease Control (CDC) Social Vulnerability Index (SVI) census tracts.
Justice40 (FEMA Now)	Census tract	Allocate budget to households based on highest to lowest net benefit from elevating a structure to its economically optimal elevation height. The majority of benefits must accrue across households in census tracts that the Climate and Economic Justice Screening Tool (CEJST) identifies as “disadvantaged.”

We conduct our analysis in a Gloucester City, New Jersey case study. Gloucester City is an instructive case study location because it reflects many environmentally and economically burdened small cities across the nation that are overlooked by federal grant programs. The city ranks in the top 10% of most distressed New Jersey cities (63) and the state’s new Environmental Justice Law identifies the majority of its census block groups as “overburdened”(64). Beyond economic distress and legacy pollution (65), the city struggles to manage repetitive flooding because many at-risk households are outside of the federally mapped Special Flood Hazard Area and do not have flood insurance (66). The city’s hazard mitigation plan targets $1 M in direct property-level intervention costs and a $3 M project budget to reduce flood risk for repetitive loss households (66). However, the city faces resource and capacity challenges in identifying properties to prioritize and preparing grant applications (66, 67).

To address our research questions, a detailed local case study is preferable to a coarse larger-scale study for several reasons. First, our research questions require a very fine-scaled analysis because flood-risk varies at fine scales and funding prioritization based on “disadvantaged community” status may not capture which households will benefit from elevation in either an economic or equity perspective (18, 20, 58). This requires a high-resolution hazard modeling approach that is not easily applied at large scales due to prohibitive computational expense (68–71). Second, a local case study fulfills the need to investigate place-specific adaptation success in the context of broader policies that shape local outcomes (8, 72–75).

## Results

### Spatial Distribution of Risk Burden.

We assess the spatial overlap of household flood-risk burden and four definitions of “disadvantaged community” to evaluate the potential implications of spatial mismatch between community-level prioritization and household-level equity objectives. First, we consider CEJST “Justice40 Communities” as used by FEMA’s Justice40-covered programs to prioritize household elevation funding (11, 12, 42). CEJST identifies these “disadvantaged communities” as census tracts above the 65th percentile of all US census tracts for percent of households with income at or below 200% of the federal poverty level indicator *and* at or above the threshold in at least one additional burden category (52). Second, also defined at the census tract level, is the Center for Disease Control (CDC) Social Vulnerability Index (SVI) (76). FEMA used this definition in 2021 and 2022 to prioritize funding from the above programs (77–79). Third, defined at the block group level, is the Federal Housing Administration (FHA) low- and moderate-income census block groups which indicate prioritization areas for Census Development Block Group Disaster Relief (CDBG-DR) grants that can fund flood-risk reduction activities (42, 80–82). Fourth, also at the block group level, is New Jersey overburdened communities, recently defined in New Jersey state law to identify communities “in need of environmental justice (64).” Notably, FEMA also uses the new Community Disaster Resilience Zones to prioritize communities for funding under some programs (11, 12), but no census tracts in our case study qualify for this definition.

**Fig. 2.**
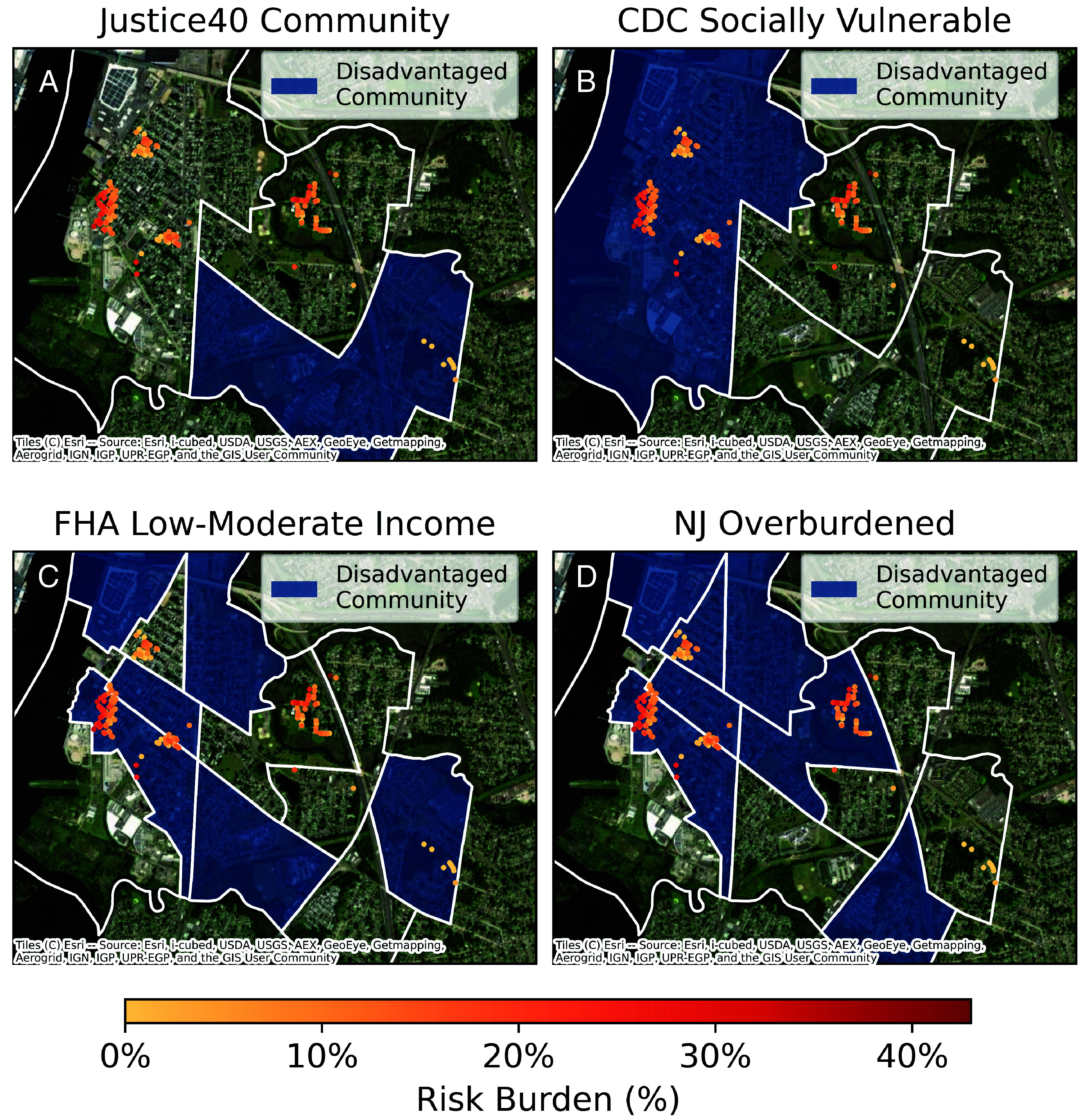
“Disadvantaged communities” and the spatial distribution of flood-risk burden. Each panel shows the same spatial distribution of risk burden. Census tract (block group) boundaries are delineated by white boundary lines in the *Top* (*Bottom*) panels. Across each panel, a different federal (*A*, *B*, and *C*) or state (*D*) agency “disadvantaged community” spatial dataset is plotted in blue shading. Panel *A* shades areas that correspond to Justice40 communities, as defined by the Climate and Economic Justice Screening Tool (CEJST), a FEMA prioritization criterion from 2023. Panel *B* shades areas that correspond to values of greater than 0.6 of the Center for Disease Control (CDC) Social Vulnerability Index, a Federal Emergency Management Agency (FEMA) funding prioritization criterion from 2022. Panel *C* shades areas that correspond to majority low- and moderate-income households, according to the Federal Housing Administration low- and moderate-income block groups data, a prioritization criterion for the Community Development Block Grant (CDBG) program. Panel *D* shades areas that correspond to New Jersey (NJ) overburdened census block groups, a criterion that NJ currently uses to identify communities in accordance with NJ Environmental Justice law.

Applying all “disadvantaged community” definitions shows that there are communities worth prioritizing in Gloucester City. However, the conclusions on which areas to focus on differ (Fig. 2). Notably, CEJST identifies one tract as “disadvantaged.” This tract ranks at the 65th percentile for the low-income indicator and the 90th percentile for one transportation and one pollution burden (65). However, it ranks at the 20th percentile for the flood burden metric, which corresponds to the few risk-burdened households in its boundaries. In contrast, the census tract that exceeds the CDC SVI threshold captures far more risk-burdened households, ranks at the 70th percentile for flood burden, and is the only tract in Gloucester City that experienced neighborhood redlining (65). This tract is not “disadvantaged” according to CEJST because it ranks at the 59th percentile for the low-income indicator. However, CEJST classifies this and the other non-“disadvantaged” census tract as ranking higher in other economic burdens and as burdened across more indicators than the Justice40 community (*SI Appendix*, Table S1).

In general, these coarsely defined prioritization areas poorly capture households with high flood-risk burden in Gloucester City (Fig. 2 and *SI Appendix*, Figs. S1 and S2). The one “Justice40 Community” contains only a few houses that face flood risk and captures none of the households with relatively high-risk burden. Although the three other definitions capture flood-risk burden better than CEJST, they still exclude some areas with high-risk-burdened households (Fig. 2 and *SI Appendix*, Figs. S1 and S2). For instance, New Jersey’s overburdened communities definition captures nearly all households with risk burden, but like all the definitions considered here it excludes the most risk-burdened property.

### Targeting Households Most in Need.

We design and test rules that prioritize funds based on different ways of defining “disadvantaged communities” to evaluate how well community funding rules target households with high-risk burden. To be consistent with current funding rules, we implement the majority of benefits in a “disadvantaged community” constraint (11, 12, 82). We sort households in terms of net benefit from their optimal elevation heightening subject to the constraint that the majority of benefits must accrue within the relevant “disadvantaged community” (*SI Appendix*). We show results using New Jersey overburdened communities, which are not currently used to prioritize funding, in *SI Appendix*.

We also design and test simple funding rules that prioritize funding across households. The first household rule prioritizes households based on highest to lowest net benefit from their optimal elevation. The second prioritizes households based on highest to lowest reduction in risk burden from optimal elevation. The third prioritizes households in terms of highest to lowest initial risk burden.

**Fig. 3.**
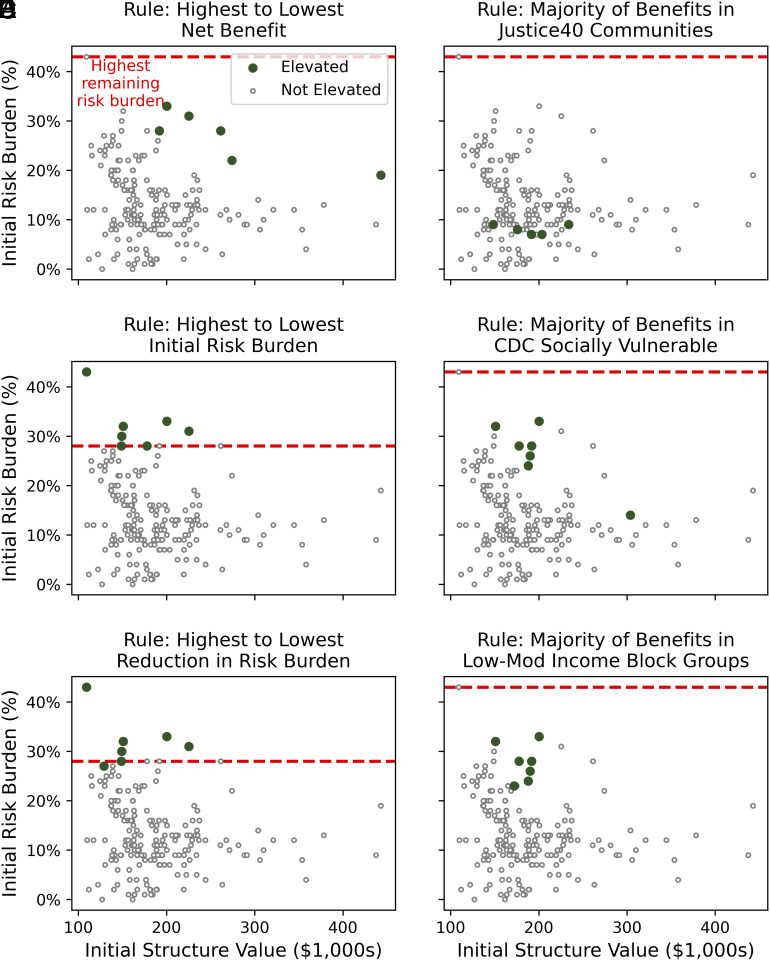
The distributional implications of considered funding rules under a $1 M allocation budget. Points represent individual structures in terms of their structure value and flood risk as a percentage of that value. Each panel shows the results of implementing a funding rule for a $1 M budget (roughly the 75th percentile of all FEMA Hazard Mitigation Assistance flood elevation grants in 2022 dollars). Panels *A*–*C* show household rules and panels *D*–*F* show community rules. Houses receiving funding under each rule are plotted in green. Houses shown in gray do not receive funding. The dashed red line indicates the highest remaining risk burden after elevating houses, a quantity that indicates a more equitable investment when it is lower on the y-axis.

Prioritizing funding based on net benefit can be ineffective at targeting some households with relatively high-risk burden, especially when the majority of benefits must accrue in a “disadvantaged community.” For example, under a $1 M budget, rules that prioritize funding based on net benefit do not allocate funds to the most risk-burdened households (Fig. 3 and *SI Appendix*, Figs. S3 and S4). In fact, the household rule based on net benefit recommends elevating the most valuable household, but not the most burdened household—even though the former has less than half the risk burden (19% versus 43%) and more than four times the structure value ($443 K versus $109 K) of the latter (Fig. 3). This is a decision-relevant budget because it is the amount of funding Gloucester City seeks for property-level interventions in its hazard mitigation plan (66). Only after nearly tripling the budget to $2.5 M do community-level rules prioritize this most burdened household (*SI Appendix*, Figs. S5 and S6).

FEMA’s current Justice40 implementation especially fails to target households with high-risk burden in this case study (Fig. 3*D*). This is because the cost to elevate these houses exceeds the benefits from elevating them. In this case, the constraint that the majority of benefits must accrue in this census tract makes it infeasible to allocate funds for a household with a high-risk burden. This illustrates how the current FEMA implementation of the Justice40 Initiative can introduce funding obstacles for municipalities that seek to prioritize this conception of equity across households.

### The Cost of Reducing Risk-Burden Inequality.

**Fig. 4.**
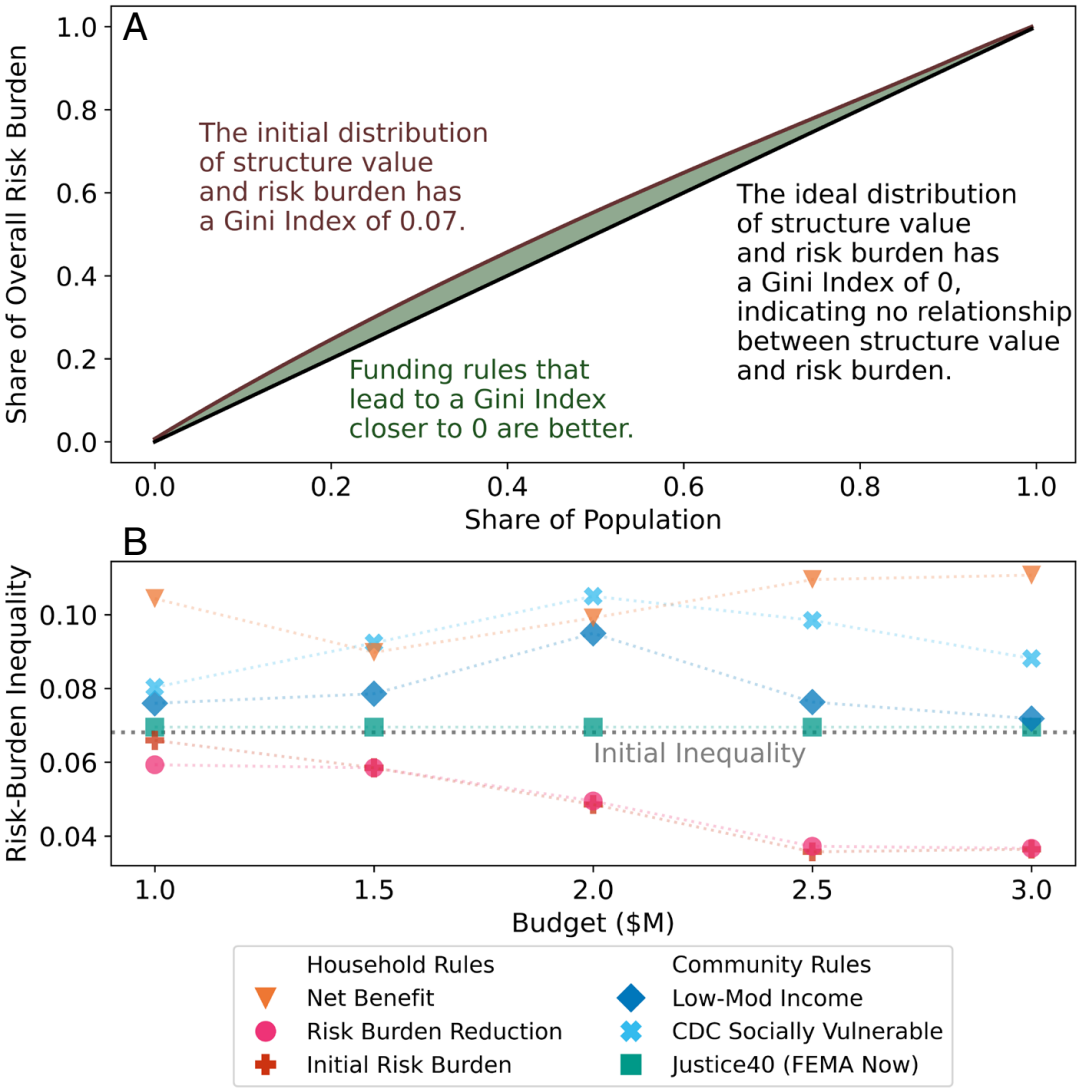
The cost of reducing risk-burden inequality. Panel *A* shows the initial distribution of risk-burden inequality for properties sorted by structure value. Panel *B* shows the risk-burden inequality (unitless from 0 to 1) obtained by different funding rules under a variety of project budgets. A gray dashed line shows the initial inequality to contextualize which rules worsen or lessen inequality for different project costs. Note that objectives are only evaluated at each displayed point, but dashed lines are shown to enhance visual comparability.

Only funding rules based on risk burden reduce risk-burden inequality under decision-relevant budgets (Fig. 4). Based on Gloucester City’s hazard mitigation plan (66), decision-relevant budgets range from $1 M to $3 M, respectively corresponding to the city’s targeted flood mitigation investment costs and overall budget (e.g., including administration costs). The initial share of risk burden is slightly more concentrated in lower-value households (Fig. 4*A*). Under a $1 M budget for investment costs, the rules based on risk burden slightly reduce inequality. At a $3 M budget for investment costs, these rules continue to decrease inequality in residual risk burden. Notably, a $3 M budget corresponds to roughly the 90th percentile of inflation-adjusted FEMA elevation grants (*SI Appendix*), reflecting an unlikely award size for a small, underresourced municipality such as Gloucester City (50, 83, 84).

In contrast, rules based on net benefit increase risk-burden inequality in this decision-relevant budget range (Fig. 4 and *SI Appendix*, Fig. S7). The allocations based on FEMA’s implementation of the Justice40 Initiative only slightly increase risk-burden inequality. In comparison, the other rules based on net benefit induce higher levels of inequality under a wide range of considered budgets. These rules only reduce inequality at budgets over $6 M, which corresponds to nearly the 95th percentile of inflation-adjusted FEMA elevation project costs (*SI Appendix*, Fig. S8).

Importantly, increasing the budget can increase risk-burden inequality, regardless of the funding rule (Fig. 4). For example, the CDC SVI community rule results in higher inequality under a $2 M budget than $1 M budget. Similarly, sorting by household net benefit results in higher inequality under a $3 M budget than $1 M budget. This finding reflects that it can be difficult for decision-makers to manage concerns about redistribution of residual risk. As such, it may be helpful for decision-makers to benchmark residual risk-burden inequality to the initial level in a municipality since increased budgets can improve on other management objectives.

### Promoting Equity-Efficiency Synergies.

**Fig. 5.**
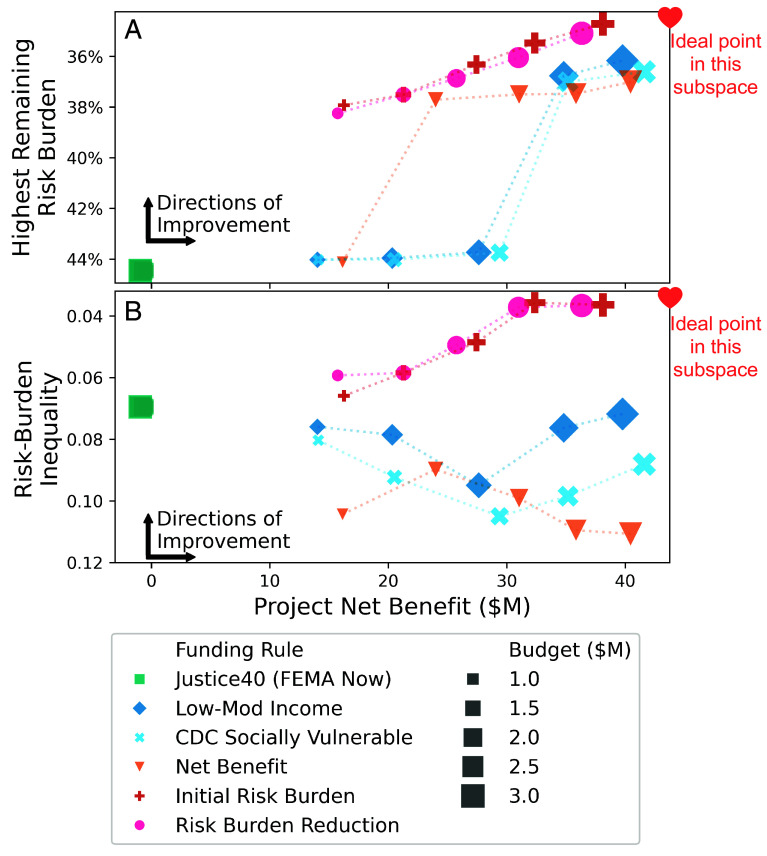
Performance of the funding rules across equity and efficiency objectives. Panel *A* and *B* shows the performance of funding rules in maximizing project net benefit and minimizing the highest remaining risk burden (risk-burden inequality). The axes are ordered such that the ideal point in each panel is in the *Upper Right* corner. Note that objectives are only evaluated at each displayed point, but dashed lines are shown to enhance visual comparability.

Simple rules based on risk burden can help decision-makers meet both equity and economic objectives. We find that these rules promote synergies between minimizing the highest remaining risk burden and maximizing project net benefit (Fig. 5*A*). In addition, these rules perform best on both minimizing the highest remaining risk burden and minimizing risk-burden inequality under decision-relevant budgets (Fig. 5*B*). Importantly, the rules based on risk burden perform similarly to rules based on net benefit in accruing overall project benefits (Fig. 5 and *SI Appendix*, Figs. S9 and S10).

Notably, community-level rules based on net benefit can promote synergies between minimizing highest remaining risk burden and maximizing project net benefit at large enough budgets (Fig. 5*A* and *SI Appendix*, Fig. S10). However, they do not at several decision-relevant budgets. We emphasize that even $1 M grants are difficult for small, underresourced municipalities to obtain in competitive programs such as those considered here (50, 83, 84). In addition, community-level rules do not reduce risk-burden inequality below initial levels for decision-relevant budgets. Further, FEMA’s implementation of the Justice40 Initiative is an obstacle to both equity and economic objectives in this case study, regardless of the budget (Fig. 5).

## Discussion

Our results suggest two overlapping reasons as to why FEMA’s implementation of the Justice40 Initiative can lead to poor equity outcomes for decision-relevant budgets. First, there is a scale mismatch between community-level prioritization and household-level equity objectives. Regardless of how one defines “disadvantage,” prioritization at the census block group or tract level can be too coarse to capture the associated fine-scaled burdens (Fig. 2) and match funding to the households in need under limited budgets (Figs. 3–5).

Second, there is a definition mismatch between prioritization and equity objectives. We defined equity objectives for the specific goals of the FEMA Justice40-covered programs and corresponding CEJST burden indicators. As such, we defined our equity objectives at the household scale and based on notions in environmental justice about risk burden. The other community rules are grounded in related, but distinct, concepts of social vulnerability and low income. This contributes to the definitions’ inconsistency in labeling areas as “disadvantaged” (Fig. 2). Further, since these definitions are not grounded in program-specific notions of environmental and economic burden, they can struggle to capture risk-burdened households (Fig. 2). This is demonstrated in the case study by the observation that the rule based on household net benefit increases risk-burden inequality under all decision-relevant budgets and nearly all inflation-adjusted historic budgets (Fig. 4 and *SI Appendix*, Fig. S8).

In terms of FEMA’s implementation of the Justice40 Initiative, the scale and definition mismatches overlap and can hinder a municipality’s ability to promote equity across households. Community-level prioritization requires decision-makers to make subjective and sensitive choices about which combinations of burden categories, aggregation procedures, and spatial scale determine “disadvantage.” The pitfalls of this approach are embodied by the CEJST’s poor performance in our case study. Even though there are CEJST burden categories that directly correspond to our equity objectives, this criterion performs worst. This is the case even though all census tracts in Gloucester City exceed CEJST thresholds in multiple burden categories. Further, the tract that ranks highest in burden categories relevant to the FEMA programs considered here, such as historic neighborhood redlining and high flood risk, is not “disadvantaged” according to CEJST (*SI Appendix*, Table S1).

Our results have direct implications for the design of policies that aim to equitably and economically efficiently allocate limited adaptation funds. Most importantly, policies that prioritize funding based on “disadvantaged community” status may not effectively target risk-burdened households if the way a “disadvantaged community” is defined does not capture program-relevant notions of equity. Crucially, even if a “disadvantaged community” definition does capture households in need, FEMA’s current focus on prioritizing the majority of benefits in those communities can still overlook the most risk-burdened households and increase risk-burden inequality.

Our results suggest two potential opportunities for overcoming present limitations in FEMA’s approach to implementing Justice40. First, moving from the present one-size-fits-all approach to one that empowers grant applicants to use their own preferred prioritization approach can help FEMA promote equity in local outcomes (85). This could be particularly helpful when household rules, such as those considered here, are difficult to apply due to data and resource limitations. For instance, decision-makers could readily prioritize funds based on New Jersey overburdened communities, which performs better on all objectives than the current Justice40 approach in our case study (*SI Appendix*, Figs. S21–S23). If FEMA and federal agencies focus on standards for funding prioritization rules [e.g., perhaps drawing from a recent National Academies report on valid geospatial tools for environmental justice (26)], it can give communities the needed autonomy to meet local preferences for equity (85).

Second, interagency coordination and the collaborative public provision of climate services could help the federal government provide needed high-resolution risk metrics for decision-makers to achieve equity goals (86, 87). Many federal agencies develop and procure data that provide inputs for flood-risk analyses, such as high-resolution topographic and bathymetric data (87–89). We echo calls for a national framework for regional collaborative flood modeling to support the production of actionable flood hazard data products nationwide (87). Our risk estimation can complement this effort and enable a range of end users to evaluate prioritization criteria and metrics that meet their notions of equity. Beyond data that support prospective analyses of flood risk and associated inequities, widespread procurement of data on historical flood impacts and environmental burdens can support efforts to prioritize households in need (49).

Our study is subject to several caveats that point to research needs. First, our case study is defined by a relatively small municipality with most households in some form of a “disadvantaged community” (Fig. 2). This reduces the generalizability of our findings to the national scope and cross-agency coverage of the Justice40 Initiative. To date, it is computationally challenging to implement our framework at much broader scales. Considering larger spatial scales (for example, across the United States to match the scope of the Justice40 Initiative) could provide fundamental insights about scale mismatch implications and the nature and magnitude of equity-efficiency synergies and tradeoffs. For example, the reported equity-efficiency synergies may change when considering larger municipalities with more diverse exposure to environmental burdens and distinct socioeconomic stresses. In addition, it remains an open question as to which locations and contexts FEMA’s implementation of the Justice40 Initiative helps decision-makers target households in a way that best matches their conception of equity.

Second, it is important, albeit challenging, to facilitate stakeholder input about problem framing to codesign more relevant and useful analyses. For example, inputs from federal and local decision-makers, including households, can lead to more salient equity objectives (8, 90–92). In addition, these decision-makers can highlight other rules associated with grant programs that could be useful to evaluate to improve policy design and outcomes. It is an open question as to whether the rules considered here will perform well for other conceptions of equity. For example, our analysis does not consider distributional weighting, different perspectives on equity within versus between communities, or procedural and recognition notions of equity (8, 28, 44, 47, 50, 83). Notwithstanding this limitation, our results speak strongly to pitfalls with FEMA’s implementation of the Justice40 Initiative in meeting stated program goals.

Finally, we highlight potential technical refinements. First, our analysis could benefit from improved uncertainty characterization of several system components and a more sophisticated representation of compound flood hazards (93–96). Second, applying our framework on a more refined representation of the built environment (e.g., reducing bias in the location of structures and their characteristics such as first-floor elevation) could make the results more actionable for decision-makers (92, 96, 97). Third, to increase the robustness of the conclusions as well as the considered strategies, it is important to consider climate change impacts on flood hazards and important human system dynamics such as migration, social institutions, household willingness to accept funding offers, and the coevolution of risks and hazards (98–104).

In summary, we identify pitfalls in FEMA’s implementation of the Justice40 approach and identify simple funding rules that can improve on current equity and economic outcomes from climate adaptation grants. We explicate strategies that can help FEMA and other federal agencies refine their implementation of the Justice40 Initiative and better deliver on stated equity goals.

In closing, we emphasize that although the federal government’s ambitious equity goals are not trivial to meet, insights such as ours are only possible because agencies now center equity as a management objective. This paradigm shift can facilitate research inquiries that advance fundamental knowledge about equity. More importantly, it may accelerate the coproduction of knowledge between practitioners, researchers, and all constituents to meaningfully improve equity.

## Materials and Methods

We designed and operationalized a framework for analyzing decisions about which households to prioritize for funding from budget-restricted climate adaptation grant programs. We applied this framework in the context of a FEMA program that funds house elevation to defend against flooding. Although our study does not evaluate robustness, we drew from the Many-Objective Robust Decision-making (MORDM) approach to design our framework (58, 105).

A MORDM analysis is often summarized in terms of the exogenous uncertainties (“X”) that influence the system; the decision-maker’s actions or policy levers (“L”) under evaluation; the system relationships (“R”) between actions, uncertainties, and outcomes; and the metrics (“M”) that decision-makers measure to identify best-performing strategies on key objectives (58, 105) (see the XLRM diagram in *SI Appendix*, Fig. S11). In our case study, Gloucester City, New Jersey, we consider a centralized decision-maker (e.g., a federal agency) that operates a climate adaptation funding program that is covered by the Justice40 Initiative.

Because of the stated goals of the Justice40 Initiative (10), our main analytical goal was to evaluate different funding rules on equity and economic objectives and identify which rules perform well. To make claims about policy performance, our decision analysis was built on the following modeling chain (displayed in *SI Appendix*, Fig. S12).

First, we produced flood hazard maps, at one-meter resolution, that account for pluvial, fluvial, and coastal flooding across twelve return periods of 1, 2, 5, 10, 15, 20, 25, 50, 75, 100, 200, and 500 y. Then, using the Uncertain Structures and Fragility Ensemble (UNSAFE) framework for property-level flood-risk estimation (106), we combined these flood hazard maps with single-family houses, as represented by the widely used national structure inventory (NSI) (20, 53, 107), and estimated expected annual damages under shallow uncertainties in structure value, first-floor elevation, and depth-damage functions (DDFs). We used these estimates as a counterfactual for identifying the optimal heightening for each house in the case study. Following previous work (58), we identified each house’s optimal heightening based on the elevation that maximizes expected net present value of avoided losses under shallow uncertainty in elevation costs, elevation benefits, house lifetime, and discount rates.

After these steps, we applied the considered funding rules (Table 1) over a range of project budgets that reflect budgets for property-level flood mitigation identified in Gloucester City’s hazard mitigation plan (66). These rules identify the subset of houses that are elevated, and then the economic and equity objectives were calculated for each rule and budget combination. We describe each module of the modeling chain summarized in *SI Appendix*, Fig. S12 in detail in *SI Appendix*.

## Supplementary Material

Appendix 01 (PDF)

## Data Availability

All data and code necessary to reproduce the results in this study are available in a persistent repository. Main analysis code is available here: https://zenodo.org/records/14261389, which includes code to download input data besides flood depth grids (108). Flood depth grids are available here: https://zenodo.org/records/14260630 (109). All interim and results data are available here: https://zenodo.org/records/14261421 (110). The main analysis, including all figures and summary statistics reported in this manuscript, was successfully reproduced on September 11, 2024, by Alexis Hudes. An earlier version of the analysis was reproduced on May 20, 2024, by Prabhat Hegde. The main analysis entails all data processing and modeling after the extreme value analysis and inundation modeling modules illustrated in the workflow (*SI Appendix*, Fig. S12).
