# Supplementary material for: Funding rules that promote equity in climate adaptation outcomes
Source: Proc Natl Acad Sci U S A. 2025 Jan 7;122(2):e2418711121. doi: 10.1073/pnas.2418711121 (PMC11745337; doi:10.1073/pnas.2418711121)
Supplement: Supplementary file 1 — Appendix 01 (PDF) [file pnas.2418711121.sapp.pdf]

The file includes:

Supplementary text

Tables S1-S4

Supplementary Figures S1-S24

References

Supplementary Text

Hazard

Our hazard representation improves overall on similar decision analyses. For example, prior analyses did not use a hydrodynamic approach(59, 104), used a simpler hydrodynamic approach(43, 105), were based on models with coarser-than-household resolution(43, 105, 106), did not account for all sources of flooding(42, 59, 104, 106–108), or considered a more limited set of design events(108).

Our model domain covers the catchments of the two creeks (Little Timber Creek and Newtown Creek) that surround Gloucester City in order to capture rainfall that occasionally drains into the city. The catchments were defined using the 14-digit hydrologic units of these creeks from the New Jersey Department of Environmental Protection (NJDEP) Bureau of GIS. The link for this and other raw datasets that do not have digital object identifiers are provided in Table S2.

We performed a statistical extreme value analysis of flood drivers in our study area to produce design events with different probabilities as boundary conditions to the Super-Fast Inundation of CoastS (SFINCS)(109) hydrodynamic model. Our approach builds on a previously published high-resolution compound flood hazard framework that was used to produce one design event(10). We apply this approach to twelve design events to accommodate economic flood risk calculations across multiple return periods(11, 12).

For the statistical extreme value analysis, we considered non-tidal residual (NTR) from the Delaware river and rainfall fields as flood drivers. We followed the method from Maduwantha et al.(13) to obtain peak over threshold extreme events samples for both NTR and bias-corrected, basin-averaged, rainfall.

For NTR, we used hourly water level data at the Philadelphia tide gauge and Philadelphia Pier 11-north tide gauge to construct a 122-year-long data set from 1901 to 2021. For rainfall, we used hourly gauge data from the Philadelphia International Airport from 1901 to 2021 and gridded data from the Analysis of Period of Record for Calibration (AORC)(14) over the catchment area from 1979 to 2021. The AORC data, which is at 4km resolution, allowed us to capture spatial heterogeneity in rainfall and the hourly data from the Philadelphia Airport allows us to bias-correct this data and estimate exceedance probabilities from a longer time series(15). We used the 18-hour rainfall accumulation based on a related bivariate analysis on compound flooding in our catchment area(13).

We found thresholds for NTR of 0.63m and 18-hr rainfall of 35.6mm based on an allowance of 5 exceedances pear year on average(16, 17). Based on the HURDAT-2 tropical cyclone track dataset from the National Hurricane Center(18), we then stratified each extreme event sample into two sets: those caused by Tropical Cyclones (TCs) and those not caused by TCs. We categorized an event as a TC if the center of circulation of a TC passed through a 350 km search distance from Gloucester City within a time window of 3 days (2 days before and 1 day after). All the other extreme events were categorized as non-TC events.

We used a generalized Pareto distribution (GPD) to fit the stratified extreme samples and we estimated annual exceedance probabilities (AEP) from the fitted GPD. Because events are classified as TC and non-TC events, the probability distributions are independent. Accordingly, for a given driver (NTR or 18-hr RF), the total non-exceedance probability (ANEP) can be expressed as:

$$ANEP_{(t)} = \left(1 - AEP_{(t)}^{TC}\right) * \left(1 - AEP_{(t)}^{non-TC}\right) \quad (1)$$

where $AEP_{(t)}^{TC}$ and $AEP_{(t)}^{non-TC}$ are the annual exceedance probabilities from the corresponding TC or non-TC sample, and (t) is an index for an exceedance probability. Accordingly, the associated return period (RP) was calculated as:

$$RP_{(t)} = \frac{1}{1 - ANEP_{(t)}} \tag{2}$$

In addition to exceedance probability, to specify a design event we generated a representative event time series. We used the observed time series of NTR (from the tide gauge) and rainfall (from AORC) during Hurricane Irene, which made landfall near Cape Lookout, North Carolina on August 27, 2011 and later caused major flooding throughout New Jersey(19), to generate a scaled "t-year" event.

For scaling the rainfall for a design event, we take the proportion of a corresponding $RP_{(t)}$ value to the 18-hr rainfall accumulation from Irene and apply the proportion to the Irene hourly time series(20). For scaling the NTR for a design event, we apply a similar approach based on the cumulative NTR value from Irene. We produced the total coastal water level time series for the design event by adding hourly tidal levels and mean sea level from the past 18.6 years (to remove any effect from the nodal cycle of the tides).

The scaled time series of total coastal water levels and rainfall for twelve return periods are boundary conditions to the hydrodynamic model. We used the SFINCS model(109). SFINCS is a raster-based flood model that takes the resolution of an input digital elevation model (DEM). We used the Coastal National Elevation Database (CoNED) from the U.S. Geological Survey, a DEM with a horizontal resolution of 1m and vertical accuracy of 10 cm(21). We aggregated this DEM to 10 m using the median in ArcGIS pro 3.2.0 in order to use the subgrid approach in SFINCS(22). This approach performs calculations at the coarser resolution and adjusts water depths based on the underlying 1m grid.

In addition to the DEM, SFINCS also requires a coastal boundary, definition of inland boundaries, and input points of rainfall forcing. For the first, we placed an open coastal boundary along the middle of the Delaware river, defined by the catchment polygons mentioned earlier. The water level boundary conditions were given as a time series and placed at the Philadelphia tide gauge. For the second, we defined inland boundaries of the catchment area as outflow boundaries to allow the water to flow outside the domain (Figure S13). For the third, we used the rainfall forcing as spatially varying fields with the same resolution as the AORC data and used SFINCS to interpolate these onto the model grid resolution.

For surface roughness, we used land cover data from the NJDEP Bureau of GIS. We converted the land classifications into manning coefficients based on guidance from the Army Corps of Engineers(23). We ran the model neglecting the advection term, and thus solving the local inertia equations. The values we used for the numerical model parameters are reported in Table S3.

We used the "v2.0.0 AlpeDHuez" version of SFINCS and ran the model simulations on an Intel(R)Core(TM)i7-8700 CPU. Notably, we did not incorporate the effects of the sewer system and infiltration in SFINCS for this case study. Following the approach in Sanders et al.(10), we simulated pluvial and coastal flooding for each return period independently and then combined them into a single "compound" flood hazard map, assigning the maximum water depth in overlapping cells.

Hazard Validation

We evaluated a variety of sources to calibrate flood extent and depth estimates. However, we found major data gaps for employing high quality calibration data. This lack of observations is

typical and a persistent challenge for inundation model calibration and validation, especially in under-resourced areas such as Gloucester City(24–27). We discuss these gaps in more detail in the next section.

Due to the lack of observed data to calibrate and validate the hazard modeling approach, we configured the SFINCS model based on local expert knowledge of locations that experienced flooding in the past and compared these with simulated floods under historic meteorologic conditions(26). We were able to make use of the following sources: (i) The FEMA Risk MAP for Camden County, (ii) a local newspaper account of building-specific inundation, (iii) a tweet with photos of flooding during a historic flood event, and (iv) geo-located inundation “hotspots” identified by the Camden County Municipal Utilities Authority (CCMUA).

First, we initialized the SFINCS model configuration to reproduce flood extents from a 2009 flood event highlighted in one of FEMA’s Risk MAP products for Camden County(28) (Figure S14). The FEMA Risk MAP program guides the process for producing regulatory binding Flood Insurance Rate Maps (FIRMs). In recent years, FEMA makes much of the preliminary and ancillary information available as non-regulatory data to support local risk planning. One of these products, the 2016 “Flood Risk Map” for Camden County, highlights a 2009 flood in Gloucester City in which three inches of rain accumulated in 24 minutes(29). Notably, this flooding occurred outside the Special Flood Hazard Area (SFHA). We found that running SFINCS without infiltration reproduced flood extents at the intersection marked with a star on the Risk Map (Figure S14). As such, we did not include infiltration in generating the flood maps.

Second, we evaluated the resulting SFINCS configuration against other sources of documented flooding. We compiled a database of local news and tweets we could find with information about flooded places from historic events in Gloucester City (available in data repository). Often, this information did not include specific locations that we could use to cross-check modeled flood extents. We found location-specific information about a flooded museum from a 2019 rainfall event and a flooded intersection from a 2020 rainfall event. In both cases, our model estimates flooding at these locations (Figures S15-S16). In addition to these checks, we compared our flood simulations to geolocated flood prone locations recently identified by the CCMUA. We compared the 10-yr return period flood map to these geo-located points to evaluate whether these flood prone locations face inundation in a relatively frequent return period. We found that the 10-yr return period flood map captures the flood prone locations (Figure S17). As a final check, our 100-yr event overlaps substantially with the SFHA, but also includes the pluvial hotspots outside the SFHA identified with other validation sources (Figure S18).

These checks support our final model configuration. However, we caution that our risk and benefit estimates rely on inundation estimates that we could not directly compare to observational data. This is a persistent challenge in studies that model infrequent design events, such as the 100-yr flood(10, 30). Because of this challenge, we followed an approach from similar studies to account for scenario-based uncertainty in the considered drivers(10, 30, 31). We repeated the main analyses in the study based on forcing the SFINCS model with the 5th and 95th bootstrapped confidence intervals of combined return levels for the drivers considered in this study. We refer to these as the “lower” and “upper” hazard scenarios.

Limited calibration data for inundation model in case study location

Gloucester City identifies reducing repetitive flooding as a top priority in its hazard mitigation plan(32). In just one flood event in 2019, the city identified 80 flooded households(32). However, we were not able to obtain a comprehensive and representative set of historic flood observations for Gloucester City. Here, we describe shortcomings in various potential validation datasets for our case study.

We first consulted datasets that contain geolocated information about historic flood extents and depths. The first source we consulted was the USGS Flood Event Viewer, which the USGS created to provide geolocated coastal and riverine highwater mark records that correspond to major storms(33). This can be a useful database for calibrating and validating inundation models but contains a relatively small record of historic events and may not contain a complete record for any given event(24, 30). We did not find Gloucester City flood events in the USGS Flood Event Viewer database. Relatedly, we did not find recent Gloucester City flood events in a database called MyCoast: New Jersey, a citizen science project for documenting local flood events(35), or the Global Flood Database (derived from coarse satellite records)(27).

Lacking high-resolution, geo-located data on flood extents and depths, we turned to data on flood damages recorded by FEMA in its National Flood Insurance Program (NFIP) and Individual Assistance (IA) claims. These datasets do not include point locations but allow analysts to evaluate aggregated statistics on exposed properties and assessed damages at the census tract or zip code level. However, these datasets are prone to selection bias because of program rules. For example, properties can only file NFIP claims if they are insured at the time of a loss, a barrier for lower income populations(37–42). In addition, properties can only file IA claims if an event leads to both a presidentially declared disaster and substantial preliminary damage assessment at the county level, a process that creates barriers for marginalized populations(1, 12, 43). Because of these rules, risk-burdened properties in under-resourced communities may be left out. Indeed, there are only 9 NFIP claims and 27 IA claims in the history of each program in Gloucester City. Unfortunately, the IA claims are only available at the zip code level and Gloucester City only has one zip code, presenting a challenge for validating high-resolution flood model outputs.

Exposure

We based our exposure representation on the United States Army Corps of Engineer's National Structure Inventory (NSI). In the NSI, structure characteristics are represented by point estimates even though values are drawn from distributions or modeled processes subject to uncertainty(6, 44–47). Neglecting uncertainties can lead to biased risk estimates and decisions based on risk estimates(1, 43, 48–50). Many property-level risk assessments in the peer-reviewed literature do not account for these uncertainties in producing risk estimates(40, 41). When assessments account for these uncertainties, the procedures tend to be heuristic and not calibrated with data(44, 51, 52).

For this case study, we took structure location, foundation type, construction material, number of stories, and square footage from the NSI as fixed. We made these choices because we assume that in the planning phase for a flood mitigation grant, a municipality would know these characteristics. We used the NSI classification code "RES1" to identify single family dwellings. We further subsetted our sample to houses with one or two stories because we employ depth-damage functions (DDFs) that account for uncertainty (see next subsection) but these are available only for one and two story residential structures. Because research shows the NSI can overstate the number of structures (53), we also screened houses based on Google maps satellite imagery checks to remove points that were clearly not residential structures (See SI).

We took first-floor elevation (in feet), ffe, and structure value (in dollars) as uncertain and generate 10,000 ensemble members for each house from the following distributions:

$$ffe_i = \begin{cases} triangular(0, .5, 1.5) & \text{if } NSI\_found_i = \text{Slab} \\ triangular(0, .5, 1.5) & \text{if } NSI\_found_i = \text{Crawl space} \\ triangular(0, 1.5, 4) & \text{if } NSI\_found_i = \text{Basement} \end{cases} \quad (3)$$

$$v_i = \begin{cases} N(NSI\_val_i, .2 * NSI\_val_i) & \text{if } v_i \geq 1 \\ 1 & otherwise \end{cases} \quad (4)$$

where NSI_found_i is the foundation type and NSI_val_i is the structure value taken as-is for a structure from the NSI, indexed by i.

We represented structure value as uncertain because it is an influential driver of economic flood damage(53) and is always subject to modeling error, whether through an expert or statistical appraisal(53–55). We assumed the structure value distribution is unbiased around the value recorded in the NSI and has a standard deviation that corresponds to values from well-calibrated statistical models of house value(37). This is likely an over-optimistic representation of NSI accuracy and precision.

Well-calibrated statistical models of structure or land value make use of a large number of characteristics to accurately estimate values(1, 43, 49). In contrast, the NSI produces structure value from a non-transparent modeling process that converts raw replacement cost data from RSMeans into single cost per square foot estimates for structures with a small subset of shared characteristics(44, 48, 56). As such, it is potentially subject to undocumented mean bias and lower precision than represented here. This source of uncertainty is often not represented in property-level flood risk assessments(49) so we included a first-order attempt to capture some decision-relevant uncertainties in this important economic flood risk driver.

Our representation included a subjective researcher decision to limit the values from the specified distribution at a lower bound of $1. The technical issue this addresses is that the specified distribution could produce negative values which will lead to illogical negative estimates of risk. Because the minimum structure value in our case study is $50,323, there is a very low probability that any draw from the distribution is less than $1. For example, for the lowest value structure in our sample, the distribution we draw from implies a 0.1% chance of a realization less than $20,000. An improvement would be to use a more sophisticated approach to variation in structure value that allowed for different degrees or shapes of variance at different house-value levels.

We represented first-floor elevation as uncertain because the level of heightening that is economically optimal for a house depends on its initial elevation. Unlike structure characteristics such as location, foundation type, number of stories, and square footage that are occasionally included in property appraiser databases, first-floor elevation is not often measured or included(57). As such, we do not think it is appropriate to assume this is known at the planning phase of an application like the other structure characteristics. The distributions we draw from are based on a previous flood risk analysis with a co-author from the NSI development team(58).

Economic damages to structures

We employed DDFs from the most recent U.S. Army Corps of Engineers (USACE) report on physical depth-damage relationships because they are the most recent DDFs and because they are defined with uncertainty bounds(59). These are also an appropriate choice for our case study because these DDFs were designed explicitly with the purpose of estimating the potential benefits of flood risk management projects, such as house elevation, in the northeast Atlantic U.S. The report defines “min,” “most likely,” and “max” damages for each combination of one or two stories and basement or no basement houses. We interpret these as coming from an expert-defined triangular probability distribution.

We implemented a flood damage estimation procedure in Python, primarily using the numpy(60), pandas(61), geopandas(51), and rasterio(6, 44, 47) packages, that expands on the standard United States Hazus flood damage estimation procedure(61) by sampling uncertainty around exposure and vulnerability characteristics. We estimated structure damages for each of 10,000

ensemble members per structure as described in the previous section. For each ensemble member, we used a unique draw from the corresponding DDF probability distribution across the inundation associated with each return period. We linearly interpolated DDFs at 0.1 foot increments. We calculated expected annual loss for each ensemble member using the adjusted trapezoidal method common in the literature(62).

To estimate the present value of this risk, we modified the approach from Zarekarizi et al.(63) for representing house lifetime and discount rates under uncertainty based on data availability of uncertain discount rate projections. For discount rates, we downloaded and ran code from a repository(64, 65) that calibrates and simulates discount rates from the random discounting model in Newell and Pizer(6, 7, 48) in a Bayesian framework over a 100 year period. We followed previous guidance and imposed a non-negativity constraint for projected discount rates(37). Each house's ensemble member with the same index was given the same draw from the house lifetime distribution. These were merged with the corresponding chain from the 10,000 simulated discount rate projections available in the dataset. Then, the discounted sum for that ensemble member was calculated over the drawn house lifetime.

We emphasize that we based our risk and benefit estimates on structure values and do not include the value of contents in a house in our analysis. We make this choice because it is unclear how to characterize uncertainty in these values. Some studies include content values as a fixed portion of structure value for estimating risks and benefits(44, 45, 49). This is also how the NSI represents content values(66). However, this is not a standard researcher decision(66). As such, the choice of how to represent this variable can be characterized as a deeply uncertain one(67). It is important, but beyond the scope of this analysis, to consider whether the decisions considered in this analysis are robust to deep uncertainties such as this.

Costs and benefits of elevation

We identified the level of heightening that maximizes a house's present value of avoided losses minus upfront cost and present value of residual risk. We expanded on a previous optimal elevation procedure(1, 2, 7, 47) by accounting for uncertainty in costs and benefits. Here, benefits are the present value of avoided losses minus upfront cost. We subtract benefits by the present value of residual risk to identify elevation levels that further reduce future household expected costs. Upfront cost is a one-time expense so was not discounted. In practice, it can take a long time for funds to be dispersed(68) so upfront costs may be staggered over a period of time, which could call for discounting. We consider this complexity beyond the scope of this analysis.

The empirical provenance of cost estimates for house elevation are weak, indicating large epistemic uncertainty. Despite this, studies tend to use point-based estimates of elevation cost(69). A relatively recent review article on the costs of flood adaptation cites two sources for house elevation costs in the U.S(70). Only one of these sources can still be accessed(71) and the estimates in this study are based on a 2009 version of the FEMA Homeowner's Guide to Retrofitting(72). The more recent version of this guide, published in 2014(57), no longer includes cost estimates for elevating a house.

Other cost estimates are available based on grey literature. The Natural Hazard Mitigation Saves report by the National Institute of Building Sciences provides an estimate of $1,300 per foot of elevation with no other information about the structure characteristics of a house(73). The 2015 USACE North Atlantic Coast Comprehensive Study report provides an estimate of $192,000 per building for eight feet of elevation, assuming a typical 1,400 square foot structure(73). After adjusting for $70,000 of fixed costs, this corresponds to $87 per square foot, in line with The FEMA 2009 report estimate of $88 per square foot for elevating a wood frame slab-on-grade house by eight feet. Lastly, the 2017 Louisiana Coastal Master Plan report(72) provides estimates for a 2,000 square foot structure without a basement based on RSMeans data, and also the same

2009 FEMA report. Appendix A(73) (p. 97, Table 16) suggests it costs $86.25 per square foot to elevate a 2,000 square foot slab-on-grade structure by seven to ten feet, also in line with the FEMA 2009 report.

We used the 2009 FEMA report as a basis for our cost estimates to be consistent with previous peer-reviewed and gray literature that informs large-scale climate adaptation plans. The report provides estimates for structures with different foundation types, construction materials, and costs per square foot at different levels of heightening. We took all these features as fixed from the NSI. We linearly interpolate to obtain unique costs for different levels of heightening, following Zarekarizi et al.(74) and Doss-Gollin and Keller(74, 75). Because fixed costs are included in the 2015 USACE report (around $70,000) and the 2017 Louisiana Coastal Master Plan (around $20,000), which includes things like permitting, temporary relocation fees, inspection, title cost, and surveying, we also included these. We focused on heightening from three to ten feet because cost estimates above ten feet are only available for one-story houses without basements in the Louisiana Coastal Master Plan report.

We sampled cost uncertainty using a simple approximation. First, for fixed costs, we uniformly sampled from $20,000 to $70,000 based on estimates described above. Second, for variable construction costs (which are scaled by a house's square footage), we adjusted the values from the 2009 report by a construction price deflator uniformly sampled between the Bureau of Labor Statistics non-seasonally adjusted index for wages and salaries for privacy industry workers in construction(75, 76) and the Laspeyres (Fixed) Census Bureau of Labor Statistics construction price indices for single family houses under construction(75).

Our approach allowed us to represent elevation costs in terms of 2022 dollars and accounts for some uncertainty due to a combination of labor and construction cost inflation. We generated 10,000 draws from these distributions based on the candidate elevation height, foundation type, and construction material of each house and linked each one of these to one of each house's ensemble members. Please see Table S5 for the elevation cost distributions for each elevation height, foundation type, and construction material combination.

We accounted for uncertainty in benefits through uncertainty in initial first-floor elevation and DDFs. Previous studies assume known first-floor elevation, do not account for uncertainty in depth-damage relationships, or are not clear about whether counterfactual damages are estimated with uncertainty in depth-damage relationships.

A limitation of our approach is that we were not able to account for potential differences in house lifetime due to the elevation counterfactual. It is likely that houses which face flooding will have a longer lifetime if they are elevated and experience less or no flooding. However, we lack data or references to draw from to specify different distributions for house lifetime. We expect that this could lead to underestimates of benefits in our case study. Nevertheless, uncertainty in house lifetime is often overlooked, and identical house lifetime irrespective of elevation is consistent with previous research(77, 78).

Assessing funding rules

We evaluated the six funding rules introduced in the section "Targeting households in need" for how well they meet the two equity objectives and one economic objective defined in the main text. The first equity objective is to minimize the highest remaining risk burden after investment. The second equity objective is to minimize the risk-burden inequality after investment. The economic objective is to maximize the overall net benefit from investment. Here, we show how the different rules are implemented and objective values are calculated for a given budget.

Policies consist of a unique budget and a funding rule combination. The three household funding rules are:

1. Sort houses from highest to lowest reduction in risk burden from elevation;
2. Sort houses from highest to lowest initial risk burden;
3. Sort houses from highest to lowest expected net benefit from elevation.

The three community rules take the form of the third household rule but ensure that the majority of benefits accrue in Justice40 communities, low-moderate income census block groups, or CDC SVI census tracts. The source and date for each data download are shown in Table S2.

It is important to note that for the CDC SVI tracts, we used a threshold of .6 which is partially consistent with guidance in the 2022 Notice of Funding Opportunities for the FEMA Flood Mitigation Assistance and Building Resilient Infrastructure and Communities grant programs(76, 77). For individual mitigation project prioritization criteria, each funding notice stated that applications with an average SVI of greater than .6 can receive up to 30 prioritization points, and applications with an average SVI of greater than .8 can receive up to 60 points(76, 77).

For simplicity, we use a single threshold as a stylistic representation of which sets of households from our case study community could receive prioritization under rules consistent with the 2022 funding programs. In our view, the more complex rules from the 2022 national competition are more relevant to account for in analyses that evaluate applications from different municipalities. Here, we focused on how prioritization criteria affect equity within a municipality, not across municipalities. While our results about equity within a municipality speak partially to the issue of equity across communities, it is also important for more focused research into the nascent topic of how well community rules considered here affect equity across municipalities(78, 79).

We used the following procedure to implement the funding rules under different budgets. Let UC_i be the expected upfront cost of elevating house *i* by its optimal elevation height. For all budgets in the set of considered budgets, $\forall B \in \mathcal{B}$, and all funding rules in the set of considered funding rules,$\forall R \in \mathcal{R}$, we identified the set of elevated houses, $\boldsymbol{Elev}_{B,R}$, with the following procedure:

1. Define a total cost variable for the policy, $TC_{B,R}$, and initialize it to 0;
2. Define an ordered set $\boldsymbol{X}^{(R)}$ that corresponds to the given funding rule;
3. Add the upfront cost of the highest ranked house in $\boldsymbol{X}^{(R)}$ to $TC_{B,R}$ and add the house to the set $\boldsymbol{Elev}_{B,R}$, as long as $TC_{B,R} <= B$;
4. If there is a budget remaining, define a new set $\boldsymbol{X}^{(R)}_{[-k]}$, where [-k] indicates that the first k elements in the set $\boldsymbol{X}^{(R)}$ are removed;
5. Repeat steps 3 and 4 until $B - TC_{B,R} < UC_{k+1}$

For the community rules, the procedure is similar but has a few notable differences because it is not possible to identify the set $\boldsymbol{X}^{(R)}$ without checking every possible combination(80). While our problem is small enough to solve this problem using existing algorithms in reasonable time, our goal was to identify simple rules that emulate existing funding rules and might be implemented in practice. We modified the procedure above as follows:

1. Define a total cost variable for the policy, $TC_{B,R}$, and initialize it to 0;
2. Define an ordered set $\boldsymbol{X}^{(R)}_c$ that ranks houses from highest to lowest net benefit for the subset of houses that meets the disadvantaged community definition, *c*, of the currently considered funding rule;
3. Repeat steps 3 and 4 from the previous procedure on $\boldsymbol{X}^{(R)}_c$ for half of the budget;
4. Define an ordered set $\boldsymbol{X'}^{(R)}_c$ that ranks houses not in $\boldsymbol{X}^{(R)}_c$ from highest to lowest net benefit;

5. Repeat steps 3 and 4 from the previous procedure on the remaining budget, as long as the majority of benefits are produced by houses in *c,* until $B - TC_{B,R} < UC_{k+1}$

This heuristic approach is more complex to implement than the household rules. This is likely one reason why programs covered by the Justice40 Initiative have only reported the percentage of project *expenditures* that flowed to disadvantaged communities in recent grant subapplicant status reports, even though the Initiative calls for 40 percent of project *benefits* to flow to disadvantaged communities(81–83). It is possible to redefine the community rules procedure in terms of the majority of expenditures. However, we introduced the approach above to remain faithful to the stated goal of the Justice40 Initiative, even though it is more complex than household rules or focusing on expenditures.

We evaluated budgets from \$1M to \$6M in increments of \$500K and \$7M to \$15M in increments of \$1M. We only evaluated budgets from \$1M to \$6M for the lower and upper hazard scenarios to reduce overall computational run time. \$6M is roughly the 95th%ile, and \$15M is roughly the 99th%ile, of inflation adjusted budgets of historic FEMA elevation projects (See SI), so we sampled more often in the range of \$1M to \$6M. We start at \$1M, which is roughly the 75th%ile, because Gloucester City's hazard mitigation plan targets \$1M worth of property-level grants from FEMA programs considered in this study(83). In the main results, we show results from \$1M to \$3M because the latter is the overall budget the hazard mitigation plan targets for these investments(84). In the main text, we refer to this as the range of decision-relevant project budgets for Gloucester City.

After identifying $\boldsymbol{Elev}_{B,R}$ for all considered budgets and funding rules, we calculated the objective values. The MORDM approach calls for us to evaluate objectives across ensemble members with the same index. This means that for a given ensemble index, *j*, we use just that set of realizations across houses for evaluating an objective. Our final objective values are based on the expected value of these metrics across all ensemble indices. We used the following metrics where *i* is an index for houses and *j* is an index for ensemble members:

1. $NB_{i,j}$ is the net benefit of elevating the first floor by a house's optimal elevation height;
2. $RBR_{i,j}$ is the residual risk burden. This is associated with that optimal elevation height if a house is elevated and is the original risk burden if a house is not elevated;
3. $V_{i,j}$ is the structure value, which we do not modify if a house is elevated.

The first objective, maximize project net benefit, was calculated as:

$$E_J\left(\sum_i NB_{i,j}\right)$$
$$i \in \{1 \dots \boldsymbol{Elev}_{B,R}\}, j \in \{1 \dots J\} \qquad (5)$$

where E_J indicates the expected value over J ensemble members, which is 10,000 in this case study. The second objective, minimize the maximum residual risk burden, was calculated as:

$$E\left(\max_j RBR_{i,j}\right)$$
$$i \in \{1 \dots N\}, j \in \{1 \dots J\} \qquad (6)$$

where $\max_{j} RBR_{i,j}$ returns the maximum residual risk burden across *N* households in an ensemble index, j. The third objective, minimize risk-burden inequality, was calculated as:

$$E\left(\operatorname{Gini}_{j}(RBR_{i,j} | V_{i,j})\right),$$
$$i \in \{1 \ldots N\}, j \in \{1 \ldots J\} \tag{7}$$

where $\operatorname{Gini}_{j}(RBR_{i,j} | v_{i,j})$ is a function that calculates the Gini index of residual risk burden across N households, sorted by their structure value in ensemble member, j. We calculated the Gini index across a particular ensemble index as follows, where a value-sorted vector of RBR_i normalized by its maximum value and oriented at 0, is denoted as $\widehat{RBR}_i$, and population shares are denoted as $\hat{P}_i$ and also oriented at 0:

$$\frac{sum\left(abs\left(\left(\hat{P}_{i+1} - \hat{P}_i\right) * \left[\frac{\left(\widehat{RBR}_{i+1} + \widehat{RBR}_i\right)}{2} - \frac{\left(\hat{P}_{i+1} + \hat{P}_i\right)}{2}\right]\right)\right)}{2} \tag{8}$$

This is a trapezoidal approximation of the absolute difference between the empirical inequality curve and the reference equality curve of cumulative share of the population and cumulative share of residual risk burden.

In more familiar Gini index settings, such as calculating income inequality, analysts sort a population by the same quantity (e.g., income) for which they calculate cumulative share (e.g., share of income). This ensures that the empirical inequality curve is always under the reference equality curve. In our setting, we sorted the population by a different quantity (e.g., structure value) for which we calculated cumulative share (e.g., share of residual risk burden). This sorting can lead to under- and over- representation of share of residual risk burden at low and high portions of the cumulative population.

Our implementation differs slightly from Sanders et al.(84), a study we base our inequality metric on. Whereas their index takes values from -1 to 1, our index can only take values between 0 and 1, same as the Gini index for income or wealth inequality. The Sanders et al.(84) approach allows under- and over- representation to cancel out. In contrast, our approach represents both types of distributional inequality. This is more consistent with the corresponding equity objective's underlying distributive principle that increasing risk-burden inequality is inequitable, regardless of which end of the structure value distribution that occurs. Other distributive principles could call for a different mathematical implementation.
We conducted sensitivity analyses to evaluate the robustness of our conclusions about funding rules to uncertainties that are challenging to reconcile in our case study due to data limitations. First, because of limited validation data for flood modeling, we evaluate the funding rules under two alternative hazard scenarios. We describe this approach and the robustness of our conclusions to these other scenarios in the "*Sensitivity of results to lower and upper hazard scenarios*" section of this supplementary text below. Second, beyond conceptual disagreements, another aspect of equity being pluralistic is that the same notion of equity can be measured in different ways(85). As a sensitivity analysis, we measured the minimize highest remaining risk burden objective in three other distinct mathematical forms that seem consistent with the motivating distributive principle that lower risk burden is better. We discuss these results in the "*Sensitivity of results to uncertainty in equity objectives*" section of this supplementary text below.

Inflation-adjusted hazard mitigation assistance grant elevation project costs

We conducted a supplementary analysis on FEMA Hazard Mitigation Assistance (HMA) historic flood elevation grant project costs to contextualize the budgets considered in the main analysis. We downloaded the OpenFEMA Dataset: Hazard Mitigation Assistance Projects – v3 dataset on March 18, 2024 (Table S2). We subsetted the dataset to projects before 2023 and two project types: elevation of riverine and coastal private structures, specified by project type codes 202.1 and 202.2, respectively. We inflation adjusted project costs to 2022 dollars based on the Laspeyres (Fixed) Census Bureau of Labor Statistics construction price indices for single family houses under construction(75). We contextualized budgets based on the inflation-adjusted elevation project cost percentiles.

Sensitivity of results to uncertainty in equity objectives

As discussed above, we measured the minimize highest remaining risk burden objective in three other distinct mathematical forms that seem consistent with the motivating distributive principle that lower risk burden is better. We evaluated this objective in terms of minimizing the median, sum, and 95th%-ile of residual risk burden (Figure S19). We found that all considered rules, besides the Justice40 rules, perform similarly to each other when these other mathematical forms are taken. Generally, the rules that prioritize risk burden still perform better, as in our main specification. However, the performance gap between risk-burden and other rules is typically smaller.

While we don't measure the inequality metric different ways, our results suggest that more research is needed on when it is a good metric to use or how to use it for smaller sample sizes in a risk-reduction context. For example, the risk-burden rules induce more risk-burden inequality than any of the net benefit rules at relatively high budgets (Figure S8). Because these rules target the most risk-burdened households, this result may reflect sensitivity of the inequality metric to two features. First, there are a small number of households at risk in our main hazard scenario. Second, and relatedly, higher budgets reflect an increasingly large proportion of households with very low residual risk, inducing an increasingly higher share of risk burden for higher-valued structures. Supporting these notions, risk-burden rules reduce inequality for budgets above $3M under the more intense hazard scenario in which ten times the number of properties are at risk (Figure S7).

Analysis of New Jersey Overburdened Communities

We reproduced Figures 3, S5, 4, and 5 by replacing Justice40 communities with New Jersey overburdened communities(6). These are shown in Figures S20-S23, respectively. We evaluated New Jersey overburdened communities as supplementary results because these definitions are not currently used in funding settings.

The New Jersey overburdened communities definition has similar performance to the household net benefit rule (Figures S19-S23). This reflects that the definition captures most risk-burdened properties besides the household with the highest risk burden in the municipality (Figure 2). As such, this rule ensures a majority of benefits accrue in New Jersey overburdened communities while prioritizing the same households as the household net benefit rule. These findings show that FEMA's current Justice40 implementation can lead to worse equity outcomes than prioritizing by household risk burden even if the "disadvantaged community" definition captures most risk-burdened households. On one hand, this ensures that benefits accrue in "disadvantaged communities," but on the other, our results show there prioritizing households by risk burden can better achieve household-level equity objectives.

Sensitivity of results to lower and upper hazard scenarios

As stated above, we tested whether our results are robust to two alternative hazard scenarios. Similar to Orton et al.(31) and Sanders et al.(10), we aimed to characterize uncertainty around water levels in each return period. We estimated bootstrapped confidence intervals for the combined return levels by fitting 1,000 bootstrapped samples to generalized Pareto distributions for both non-tidal residual (NTR) and rainfall drivers (Figure S24). In the “lower” hazard scenario, we represented levels based on the 5th percentile estimate. In the “upper” hazard scenario, we represented levels based on the 95th percentile estimate. Following the same hazard analysis described above, we used these lower and upper water levels as boundary conditions to the SFINCS model for the 12 return periods considered in this study.

We find that our results are not sensitive to these alternative hazard scenarios. Our first main result is that “disadvantaged community” boundaries poorly and inconsistently capture the most risk burdened households. This is consistent under the lower (Figure S1) and upper (Figure S2) scenario, even though ten times the number of households are at risk under the latter relative to the main hazard specification. Our second main result is that FEMA’s implementation of the Justice40 Initiative, which prioritizes investments in which the majority of benefits accrue in a “disadvantaged community,” poorly target the most risk-burdened households under decision-relevant budgets. This is consistent under the lower and upper scenario under multiple budgets (Figures S3-S4). Our third main result is that only household rules based on risk burden reduce risk-burden inequality. This is consistent under the alternative hazard scenarios (Figure S7). In fact, we also find that under the “upper” scenario, the risk-burden rules reduce inequality under higher budgets to a larger degree than they do under the main specification. Finally, our findings on objective performance are consistent across hazard scenarios (Figure S9).

Supplementary Tables

Table S1 – Climate and Economic Justice Screening Tool (CEJST) rankings for Gloucester City. CEJST burden indicators for the three census tracts in Gloucester City, New Jersey. Blue highlighted cells in the three rightmost columns indicate a ranking is above the burdened threshold. In order to be overall “disadvantaged,” a census tract must exceed the burden threshold in at least one “disadvantaged” category and at least 65th percentile for the “low income” burden. Red highlighted cells in the left most column indicate the most relevant burdens for the FEMA programs evaluated in this case study.

Tract ID	34007611000	34007605100	34007605200
Disadvantaged	No	No	Yes
Low income	59th	36th	65th
Race/ethnicity breakdown	White: 72%<br>Black: 5%<br>Asian: 6%<br>Other: 3%<br>Two or more races: 3%	White: 94%<br>Other: 3%<br>Hispanic or Latino: 3%	White: 72%<br>Black: 3%<br>Asian: 7%<br>Other: 12%<br>Hispanic or Latino: 15%

	Hispanic or latino: 12%		
Projected flood risk	70th	65th	20th
Low life expectancy	98th	74th	77th
Historically high barriers to accessing home loans	Yes	No	No
Share of households making less than 80% of the area median family income and spending more than 30% of income on housing	83rd	77th	75th
Share of homes that are likely to have lead paint	93rd	92nd	79th
Formerly used defense sites	Yes	*Missing data*	*Missing data*
Proximity to hazardous waste facilities	75th	63rd	54th
Proximity to risk management plan facilities	96th	92nd	85th
Proximity to superfund sites	96th	95th	93rd
Diesel particulate matter exposure	89th	87th	87th
Traffic proximity and volume	79th	97th	97th
Underground storage tanks and releases	93rd	84th	85th
Wastewater discharge	77th	72nd	68th
Linguistic isolation	41st	59th	73rd
Low median income	72nd	55th	51st
Poverty	38th	27th	69th
Unemployment	81st	41st	51st
Percent of people ages 25 years or older whose high school education is less than a high school diploma	7%	11%	18%

Table S2 – Raw Data Sources. Data sources with digital object identifiers are cited in the main text. Those without digital object identifiers are listed here, including the date of download.

Name	Author(s)	URL	Date of download	Input to which module
National Structure Inventory	United States Army Corps of Engineers	https://nsi.sec.usace.army.mil/nsiapi/structures?fips=34007	May 18, 2024	Decision analysis
OpenFEMA Dataset: Hazard Mitigation Assistance Projects – v3	FEMA	https://www.fema.gov/openfema-data-page/hazard-mitigation-assistance-projects-v3	March 18, 2024	Decision analysis
U.S. Census block group polygons	U.S. Department of Commerce, U.S. Census Bureau, Geography Division, Spatial Data Collection and Products Branch	https://www2.census.gov/geo/tiger/TIGER2022/BG/tl_2022_34_bg.zip	May 18, 2024	Figures
U.S. Census tract polygons		https://www2.census.gov/geo/tiger/TIGER2022/BG/tl_2022_34_tract.zip	May 18, 2024	Figures
U.S. county polygons			May 18, 2024	Figures
Gloucester City, New Jersey municipal boundary polygon	U.S. Department of Commerce, U.S. Census Bureau, Geography Division, Spatial Data Collection and Products Branch	https://services3.arcgis.com/JGF6qCAQFbROcocK/ArcGIS/rest/services/CamdenCountyMunicipalLayer/FeatureServer/0	May 18, 2024	Figures
Depth-damage functions	FEMA, USACE	https://zenodo.org/records/10027236	October 20, 2023	Decision analysis
FHA Low-Moderate Income Block Groups 2011-2015 American Community Survey	Department of Housing and Uran Development	https://www.hudexchange.info/sites/onecpd/assets/File/ACS_2015_lowmod_blockgroup_all.xlsx	November 9, 2023	Decision analysis
Climate and Economic Justice Screening Tool	Council on Environmental Quality	https://static-data-screeningtool.geoplatform.gov/data-versions/1.0/data/scor	May 18, 2024	Decision analysis

		e/downloadable/1.0-communities.csv		
CDC/ATSDR Social Vulnerability Index	Centers for Disease Control and Prevention/ Agency for Toxic Substances and Disease Registry/ Geospatial Research, Analysis, and Services Program	https://svi.cdc.gov/Documents/Data/2020/csv/states/SVI_2020_US.csv	May 18, 2024	Decision analysis
New Jersey Overburdened communities	NJ Department of Environmental Protection (NJDEP)	https://www.nj.gov/dep/gis/digidownload/zips/OpenData/Govt_census_group_2022_EJ.zip	May 18, 2024	Decision analysis
Catchment areas	NJDEP Bureau of GIS	https://gisdata-njdep.opendata.arcgis.com/datasets/02599a9424254a4ea33e689941559e3c_17/explore	January 9, 2023	Inundation model
CoNED DEM	U.S. Geological Survey<br>NOAA	https://www.usgs.gov/special-topics/coastal-national-elevation-database-applications-project/data	January 9, 2023	Inundation model
Landcover	NJDEP Bureau of GIS	https://gisdata-njdep.opendata.arcgis.com/documents/njdep::land-use-land-cover-of-new-jersey-2015-download/about	January 9, 2023	Inundation model
AORC (rainfall data)	National Weather Service (NWS), National Oceanic and Atmospheric Administration (NOAA)	https://hydrology.nws.noaa.gov/aorc-historic/	August 13, 2023	Extreme value analysis
Rainfall gauge at Philadelphia international airport	National Oceanic and Atmospheric Administration (NOAA)	https://www.ncdc.noaa.gov/cdo-web/	July 20, 2023	Extreme value analysis
Hourly water level data	National Oceanic and Atmospheric	https://tidesandcurrents.noaa.gov/stations.html	November 30, 2022	Extreme value analysis

[Philadelphia (St. ID: 8545240) and Philadelphia Pier 11-north (St. ID: 8545530)]	Administration (NOAA)			
Tropical cyclone track data	The National Hurricane Center (NHC), National Oceanic and Atmospheric Administration (NOAA)	https://www.aoml.noaa.gov/hrd/hurdat/Data_Storm.html	November 30, 2022	Extreme value analysis
OpenFEMA Dataset: FIMA NFIP Redacted Claims - v2	FEMA	https://www.fema.gov/api/open/v2/FimaNfipClaims?$filter=nfipCommunityNumberCurrent%20eq%20%27340132%27	July 31, 2024	Hazard validation
OpenFEMA Dataset: FIMA NFIP Redacted Policies - v2	FEMA	https://www.fema.gov/api/open/v2/FimaNfipPolicies?filter=nfipCommunityNumberCurrent%20eq%20%27340132%27	July 31, 2024	Hazard validation
OpenFEMA Dataset: Individual Assistance Housing Registrants - Large Disasters - v1	FEMA	https://www.fema.gov/api/open/v1/IndividualsAndHouseholdsProgramValidRegistrations?$filter=damagedCity%20eq%20%27GLOUCESTER%20CITY%27	August 28, 2024	Hazard validation

Table S3 – SFINCS model parameters.

Parameter	value	Parameter	value
mmax	921	advection	0
nmax	824	baro	1
dx	10	pavbnd	101200
dy	10	gapres	101200
x0	488084.622	advlim	9999.9
y0	4412536	stopdepth	1000
rotation	0	nuvisc	0
latitude	0	depfile	sfincs.dep
tref	20110827 130000	mskfile	sfincs.msk
tstart	20110827 130000	indexfile	sfincs.ind
tstop	20110829 010000	bndfile	sfincs.bnd
tspinup	60	bzsfile	sfincs.bzs
dtout	3600	sbgfile	sfincs.sbg
dthisout	3600	manningfile	sfincs.man
dtmaxout	129600	inputformat	bin
dtrstout	0	outputformat	net
trstout	0	cdnrb	3
dtwnd	1800	cdwnd	0 28 50
alpha	0.5	cdval	0.001 0.0025 0.0015
theta	0.9	storevel	0
huthresh	0.05	storemeteo	0
manning	0.04	storecumprcp	0
manning_land	0.04	bndtype	1
manning_sea	0.02	zsfile	zs.dat
rgh_lev_land	0	zsmaxfile	zsmax.dat
zsini	0	hfile	h.dat
qinf	0	hmaxfile	hmax.dat
rhoa	1.25		
rhow	1024		

Table S4 – Elevation cost distributions. The elevation cost distributions for each foundation type, construction material, and elevation height combination in 2022 dollars. As described in the methods, FEMA provides cost estimates for windows of heightening and we linearly interpolated these estimates for each incremental foot of heightening. In this table, we show the variable cost distribution in terms of the FEMA cost estimate.

Foundation	Material	Elevation	FEMA Estimate ($)	Variable ($)	Fixed ($)
Basement	Wood	3-4 feet	29/sq. ft	Unif(29*1.41, 29*1.85)	Unif (20000, 70000)
		5-7 feet	32/sq. ft	Unif (32*1.41, 32*1.85)	
		8-10 feet	37/sq. ft	Unif (37*1.41, 37*1.85)	
	Masonry	3-4 feet	60/sq. ft	Unif (60*1.41, 60*1.85)	
		5-7 feet	63/sq. ft	Unif (63*1.41, 63*1.85)	
		8-10 feet	68/sq. ft	Unif (68*1.41, 68*1.85)	
Slab	Wood	3-4 feet	80/sq. ft	Unif (80*1.41, 80*1.85)	
		5-7 feet	83/sq. ft	Unif (83*1.41, 83*1.85)	
		8-10 feet	88/sq. ft	Unif (88*1.41, 88*1.85)	
	Masonry	3-4 feet	88/sq. ft	Unif (88*1.41, 88*1.85)	
		5-7 feet	91/sq. ft	Unif (91*1.41, 91*1.85)	
		8-10 feet	96/sq. ft	Unif (96*1.41, 96*1.85)	

Supplementary Figures

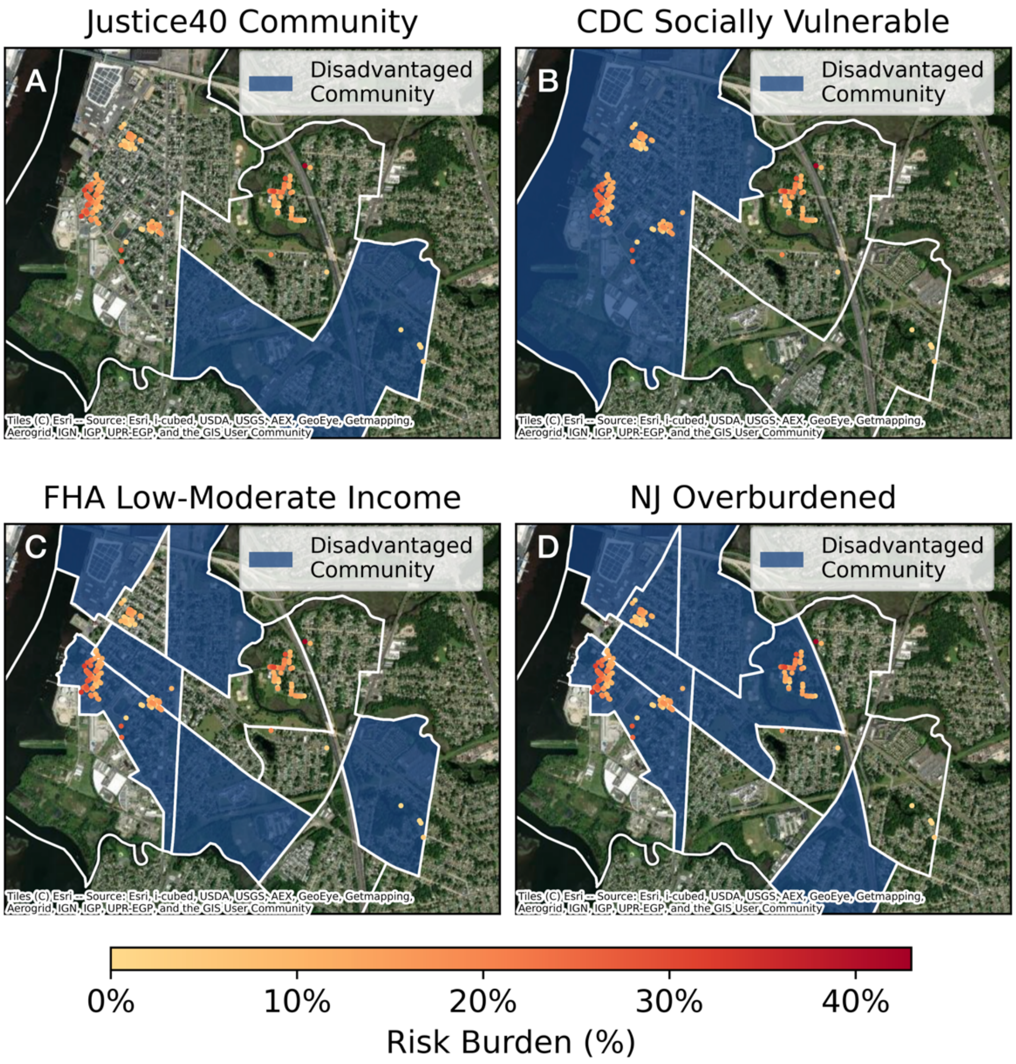

Figure S1. "Disadvantaged communities" and the spatial distribution of flood-risk burden under the "lower" hazard scenario. *Each panel shows the same spatial distribution of risk burden. Census tract (block group) boundaries are delineated by white boundary lines in the top (bottom) panels. Across each panel, a different federal (A, B, C) or state (D) agency "disadvantaged community" spatial dataset is plotted in light blue shading. Panel A shades areas that correspond to Justice40 communities, as defined by the Climate and Economic Justice Screening Tool (CEJST), a FEMA prioritization criterion from 2023. Panel B shades areas that correspond to values of greater than .6 of the Center for Disease Control (CDC) Social Vulnerability Index, a Federal Emergency Management Agency (FEMA) funding prioritization criterion from 2022. Panel C shades areas that correspond to majority low and moderate income households, according to the Federal Housing Administration low and moderate income block groups data, a prioritization criterion for the Community Development Block Grant (CDBG) program. Panel D shades areas that correspond to New Jersey (NJ) overburdened census block groups, a criterion that NJ currently uses to identify communities in accordance with NJ Environmental Justice law.*

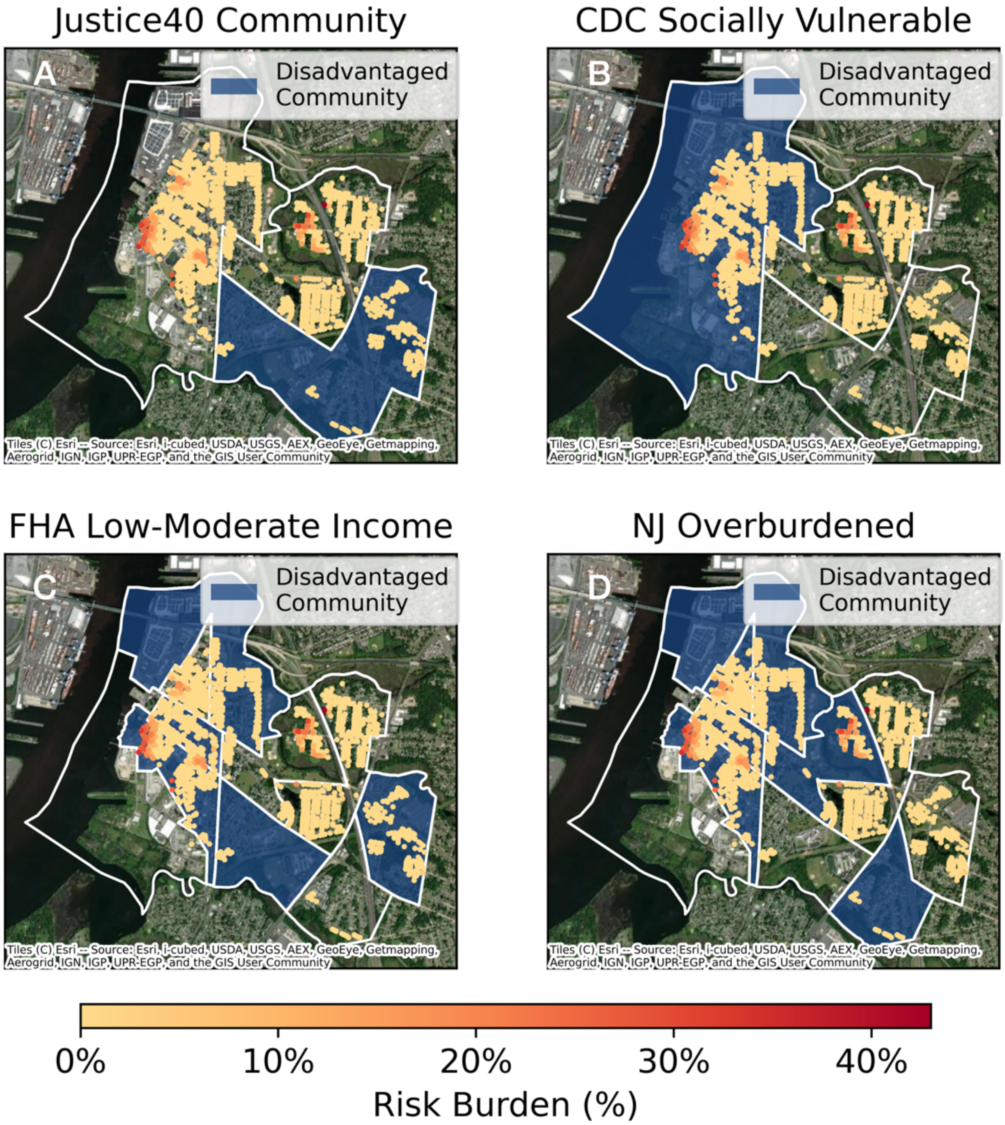

Figure S2. "Disadvantaged communities" and the spatial distribution of flood-risk burden under the "upper" hazard scenario. *Each panel shows the same spatial distribution of risk burden. Census tract (block group) boundaries are delineated by white boundary lines in the top (bottom) panels. Across each panel, a different federal (A, B, C) or state (D) agency "disadvantaged community" spatial dataset is plotted in light blue shading. Panel A shades areas that correspond to Justice40 communities, as defined by the Climate and Economic Justice Screening Tool (CEJST), a FEMA prioritization criterion from 2023. Panel B shades areas that correspond to values of greater than .6 of the Center for Disease Control (CDC) Social Vulnerability Index, a Federal Emergency Management Agency (FEMA) funding prioritization criterion from 2022. Panel C shades areas that correspond to majority low and moderate income households, according to the Federal Housing Administration low and moderate income block groups data, a prioritization criterion for the Community Development Block Grant (CDBG) program. Panel D shades areas that correspond to New Jersey (NJ) overburdened census block groups, a criterion that NJ currently uses to identify communities in accordance with NJ Environmental Justice law.*

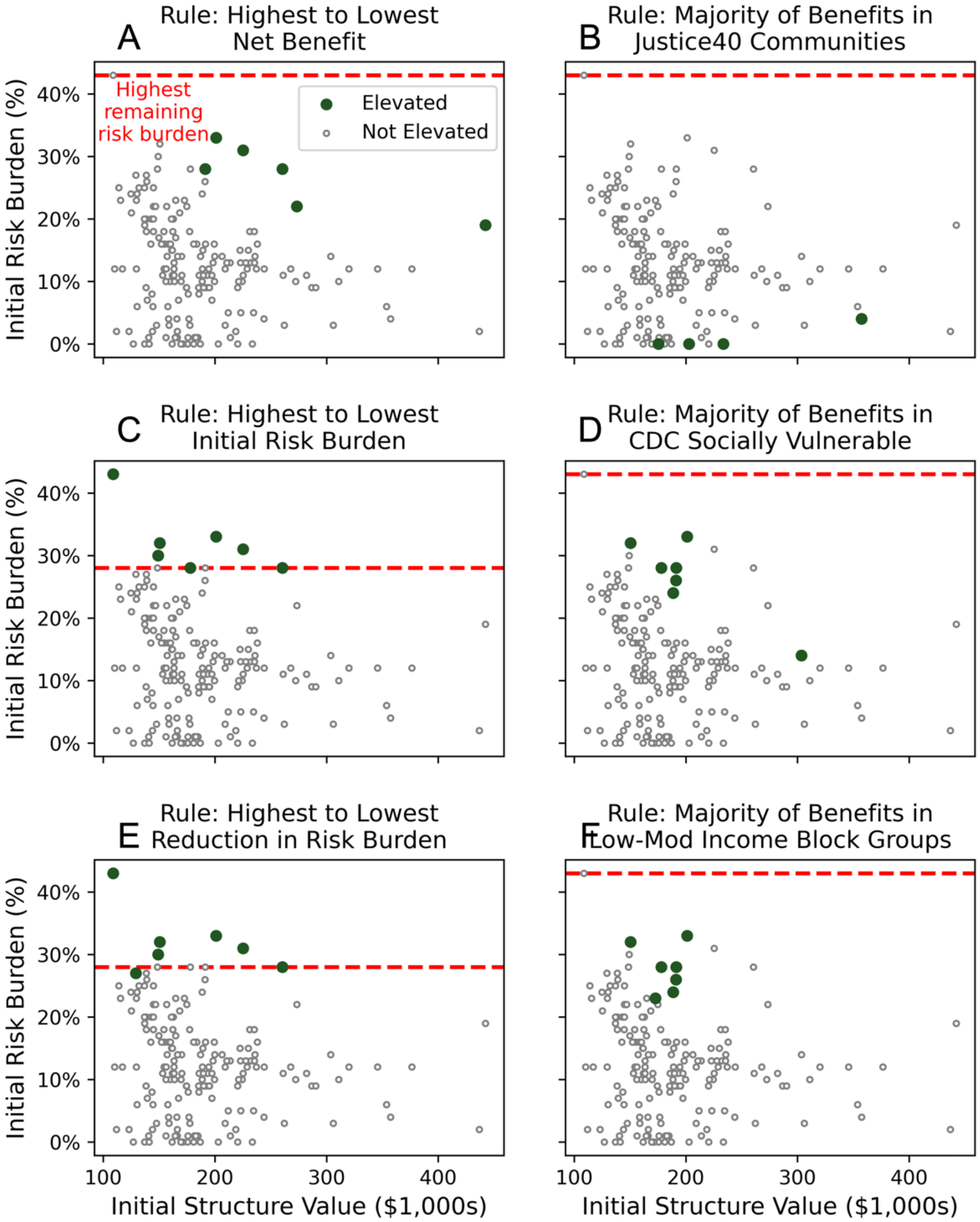

Figure S3. The distributional implications of considered funding rules under a $1M allocation budget and the "lower" hazard scenario. *Points represent individual structures in terms of their structure value and flood risk as a percentage of that value. Each panel shows the results of implementing a funding rule for a $1M budget (roughly the 75th percentile of all FEMA Hazard Mitigation Assistance flood elevation grants in 2022 dollars). Panels A, C, E (B, D, E) show household (community) rules. Houses receiving funding under each rule are plotted in green. Houses shown in gray do not receive funding. The dashed red line indicates the highest remaining risk burden after elevating houses, a quantity that indicates a more equitable investment when it is lower on the y-axis.*

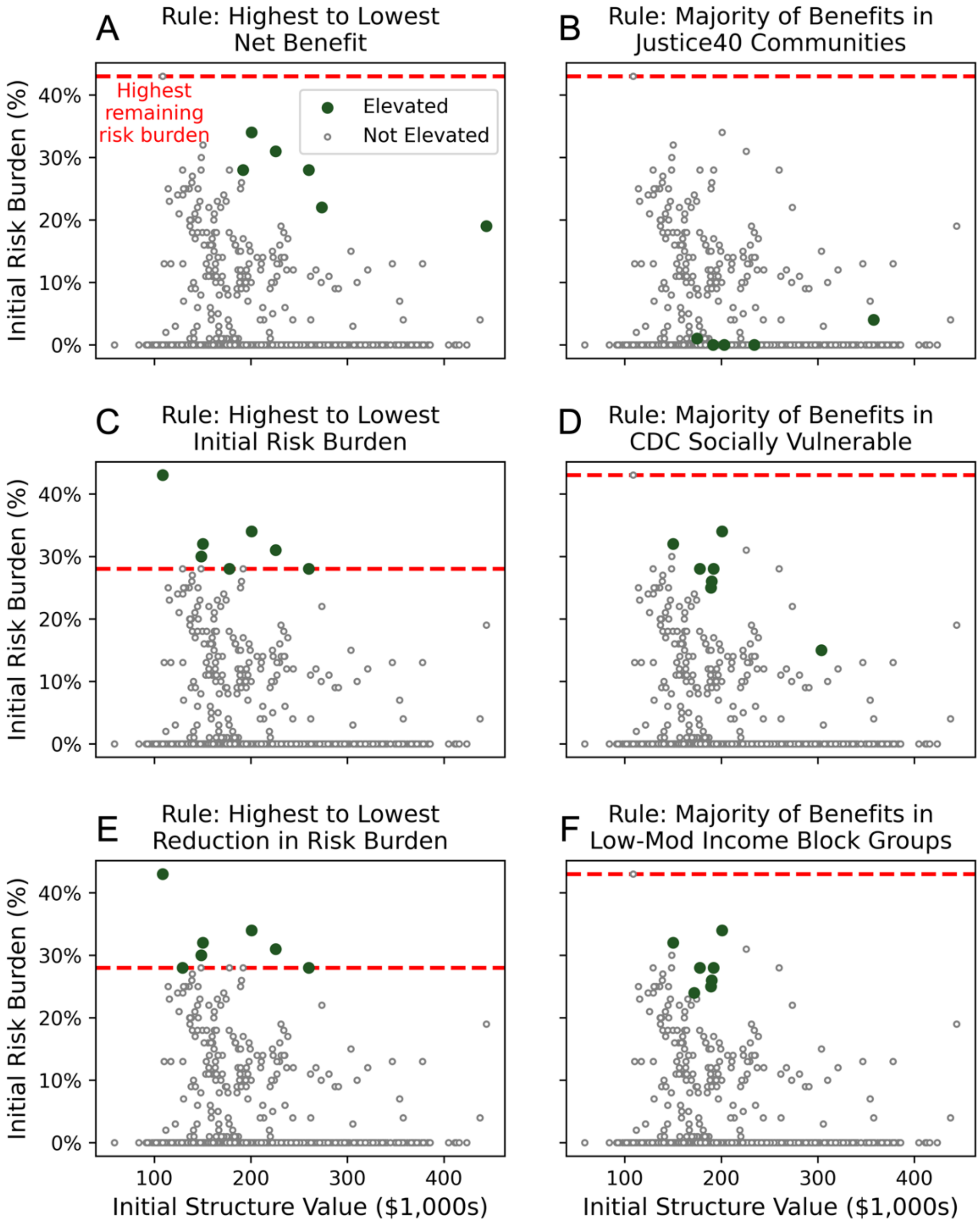

Figure S4. The distributional implications of considered funding rules under a $1M allocation budget and the "upper" hazard scenario. *Points represent individual structures in terms of their structure value and flood risk as a percentage of that value. Each panel shows the results of implementing a funding rule for a $1M budget (roughly the 75th percentile of all FEMA Hazard Mitigation Assistance flood elevation grants in 2022 dollars). Panels A, C, E (B, D, E) show household (community) rules. Houses receiving funding under each rule are plotted in green. Houses shown in gray do not receive funding. The dashed red line indicates the highest remaining risk burden after elevating houses, a quantity that indicates a more equitable investment when it is lower on the y-axis.*

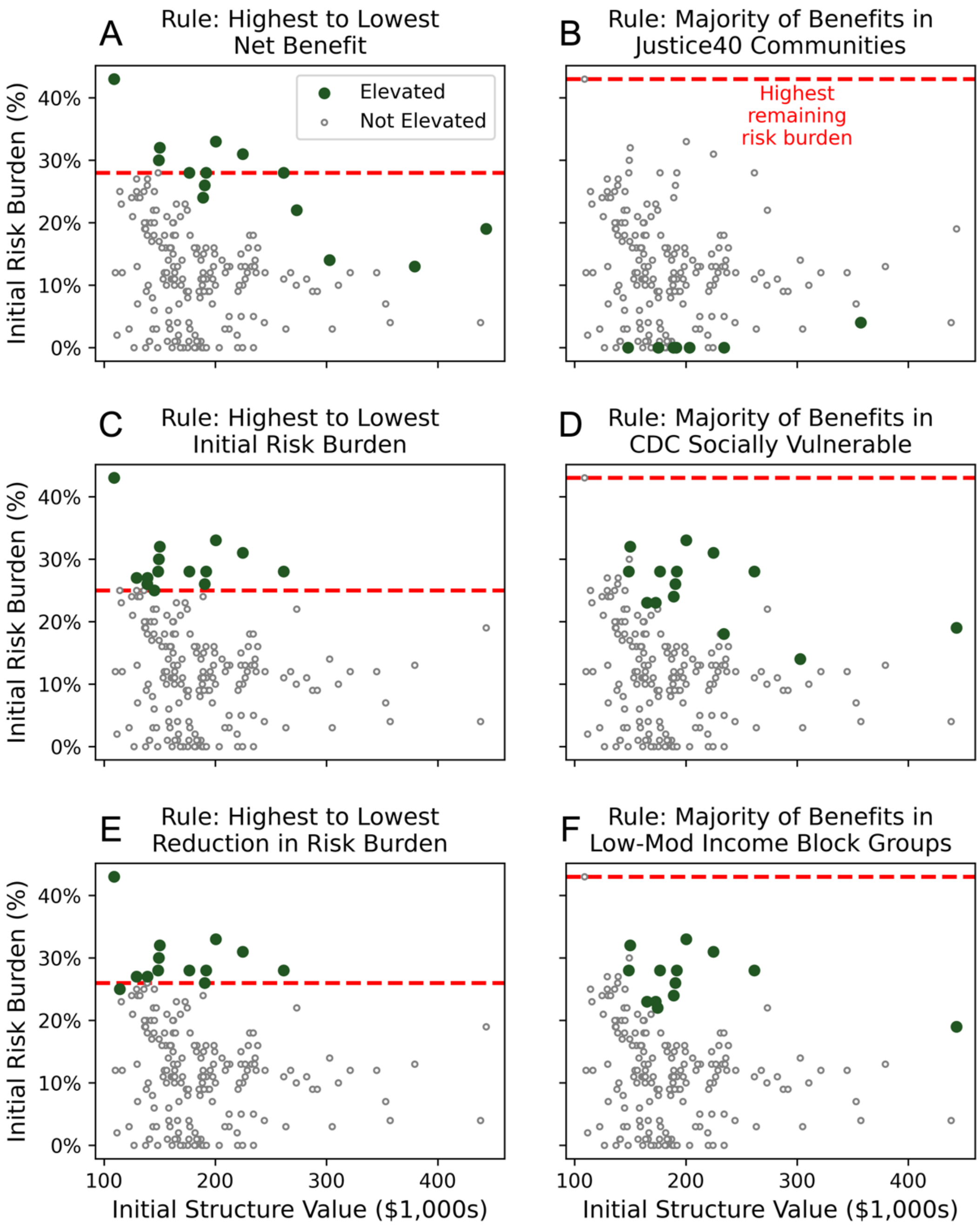

Figure S5. The distributional implications of considered funding rules under a $2M allocation budget. *Points represent individual structures in terms of their structure value and flood risk as a percentage of that value. Each panel shows the results of implementing a funding rule for a $2M budget. Panels A, C, E (B, D, E) show household (community) rules. Houses receiving funding under each rule are plotted in green. Houses shown in gray do not receive funding. The dashed red line indicates the highest remaining risk burden after elevating houses, a quantity that indicates a more equitable investment when it is lower on the y-axis.*

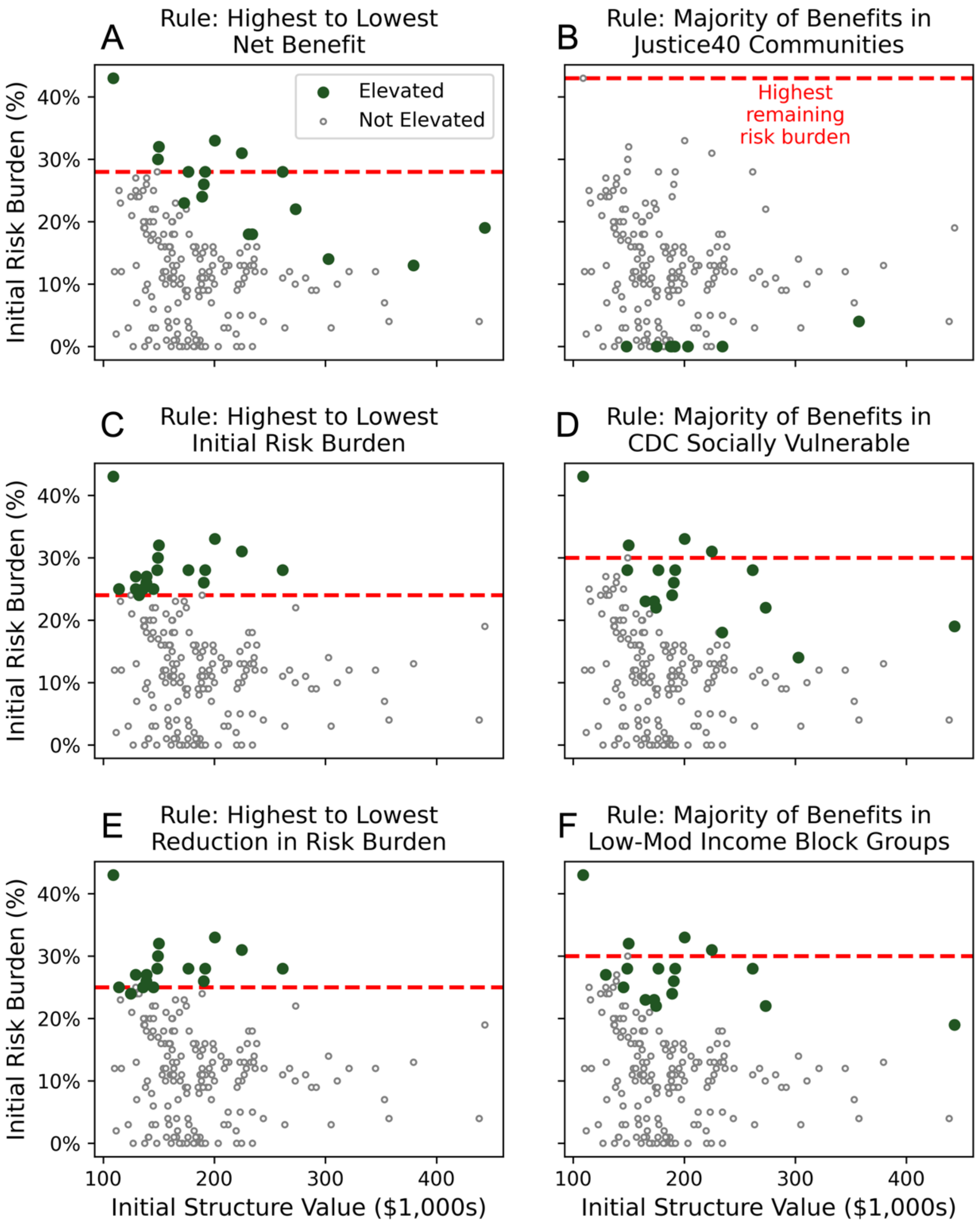

Figure S6. The distributional implications of considered funding rules under a $2.5M allocation budget. *Points represent individual structures in terms of their structure value and flood risk as a percentage of that value. Each panel shows the results of implementing a funding rule for a $2.5M budget. Panels A, C, E (B, D, E) show household (community) rules. Houses receiving funding under each rule are plotted in green. Houses shown in gray do not receive funding. The dashed red line indicates the highest remaining risk burden after elevating houses, a quantity that indicates a more equitable investment when it is lower on the y-axis.*

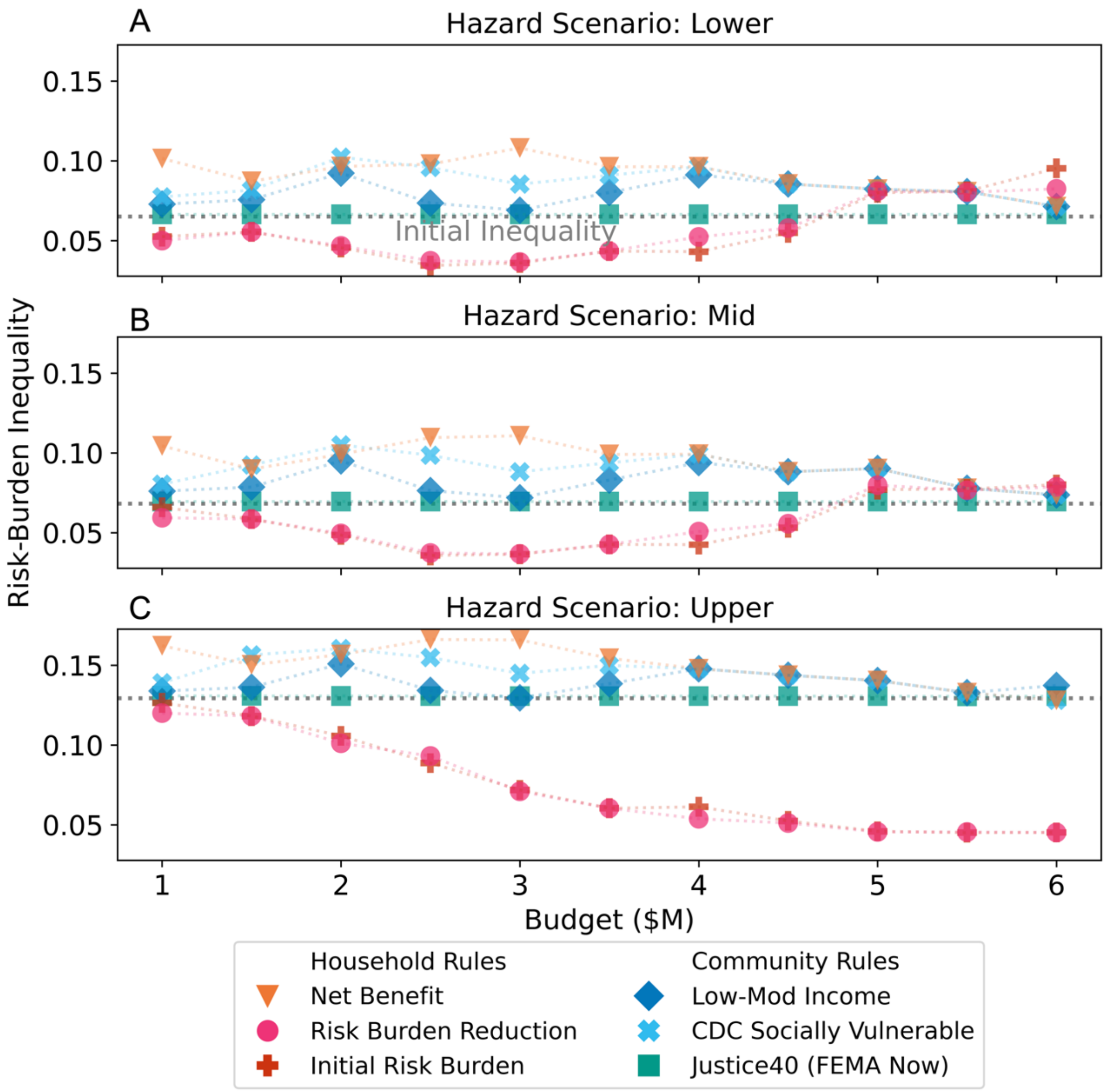

Figure S7. The cost of reducing risk-burden inequality across each hazard scenario. *Panel A shows the risk-burden inequality obtained by different funding rules under a variety of project budgets under the "lower" hazard scenario. Panels B and C show the same for the "mid" (our main specification) and "upper" scenario, respectively. A gray dashed line shows the initial inequality to contextualize which rules worsen or lessen inequality for different project costs. Note that objectives are only evaluated at each displayed point, but dashed lines are shown to enhance visual comparability.*

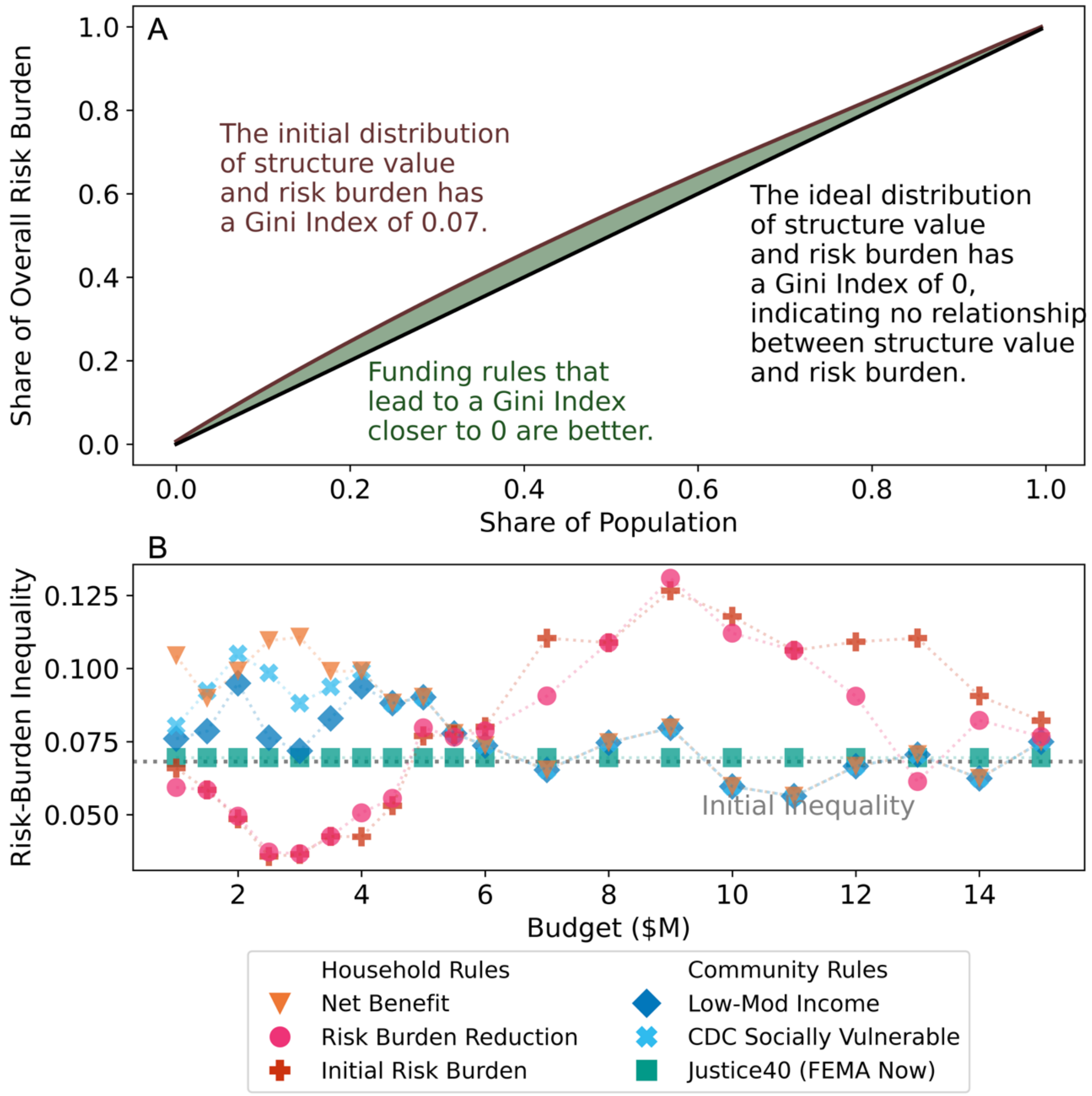

Figure S8. The cost of reducing risk-burden inequality across a wide range of inflation-adjusted FEMA elevation budgets. *Panel A shows the initial distribution of risk-burden inequality for properties sorted by structure value. Panel B shows the risk-burden inequality obtained by different funding rules under a variety of project budgets. A gray dashed line shows the initial inequality to contextualize which rules worsen or lessen inequality for different project costs. Note that objectives are only evaluated at each displayed point, but dashed lines are shown to enhance visual comparability.*

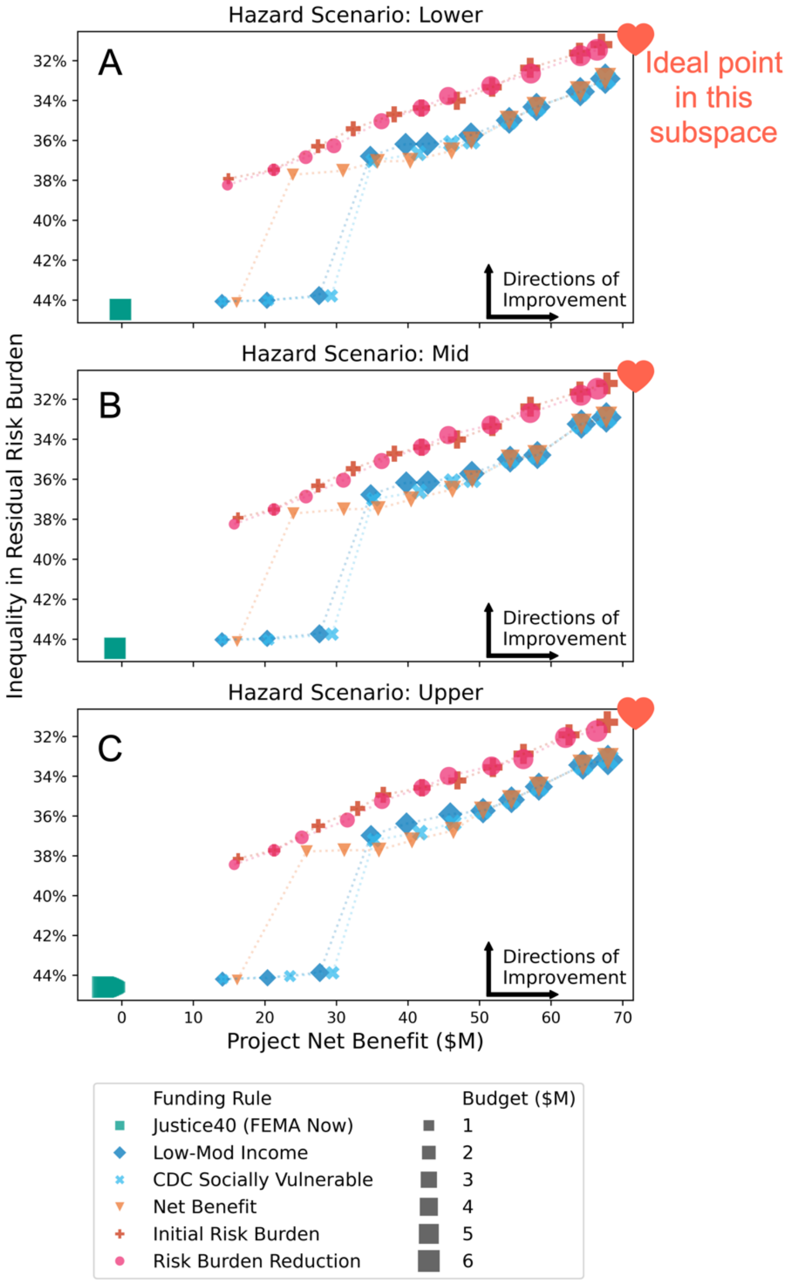

Figure S9. Performance of the funding rules across equity and efficiency objectives for each hazard scenario. *Panel A shows the performance of funding rules in maximizing project net benefit and minimizing the highest remaining risk burden under the "lower" hazard scenario. Panel B and C show the same for the "mid" (our main specification) and "upper" scenario, respectively. The axes are ordered such that the ideal point in each panel is in the upper right corner. Note that objectives are only evaluated at each displayed point, but dashed lines are shown to enhance visual comparability. Note that there is only one Justice40 point because under the considered budgets, there is only one allocation that ensures the majority of benefits accrue in the Justice40 community.*

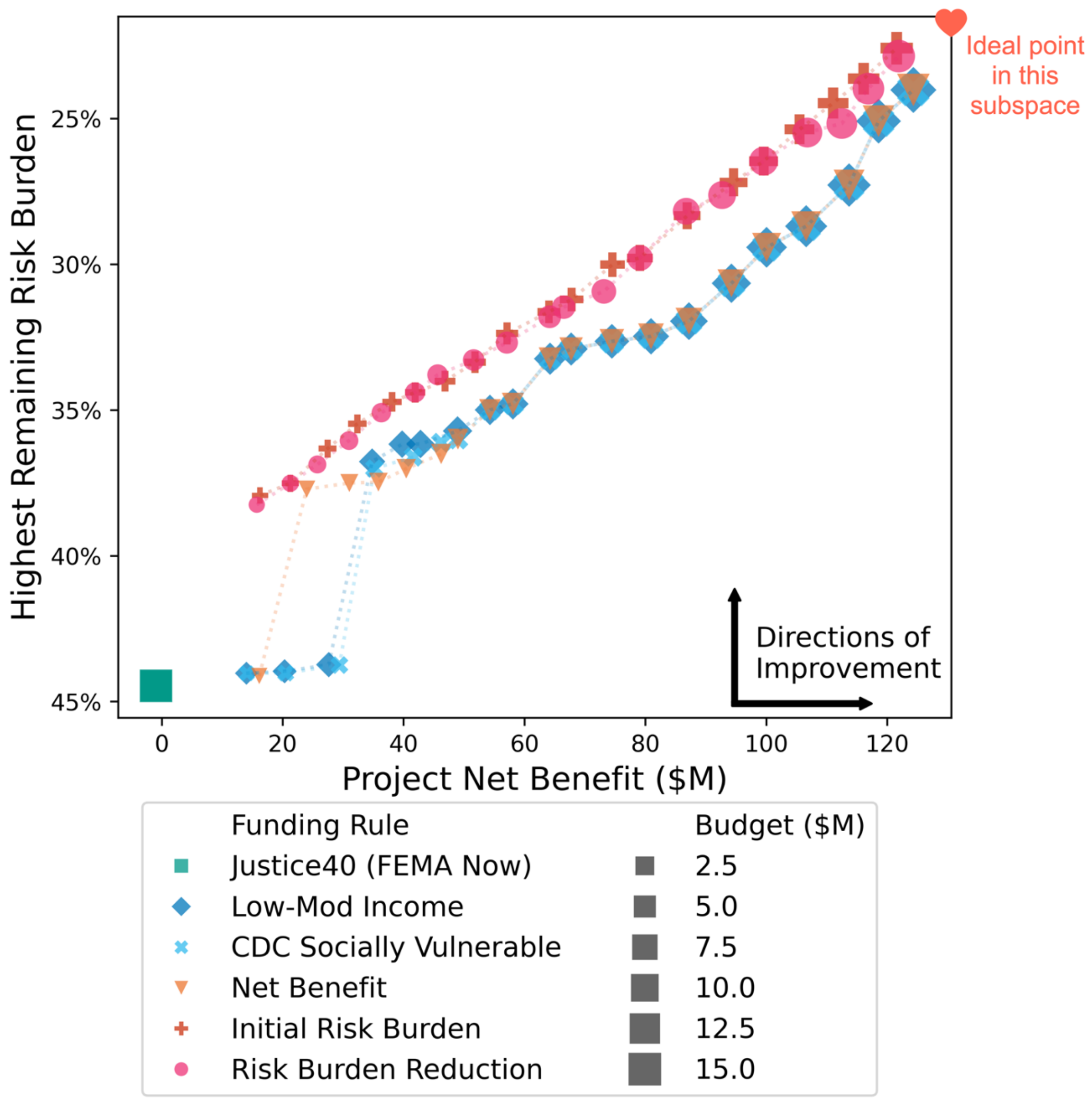

Figure S10. Performance of the funding rules across equity and efficiency objectives for a wide-range of inflation-adjusted FEMA elevation budgets. *The axes are ordered such that the ideal point in each panel is in the upper right corner. Note that objectives are only evaluated at each displayed point, but dashed lines are shown to enhance visual comparability. Note that there is only one Justice40 point because under the considered budgets, there is only one allocation that ensures the majority of benefits accrue in the Justice40 community.*

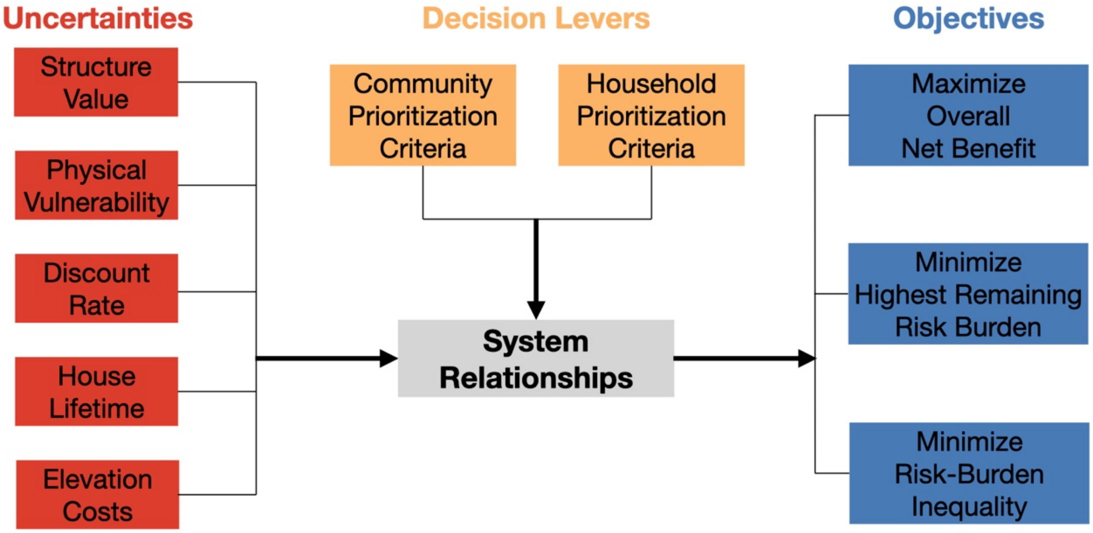

Figure S11. XLRM diagram for this case study. *This XLRM diagram represents the uncertainties (X), decision levers (L), system relationships (R), and metrics (M) expressed as objectives for this case study's many-objective decision analysis.*

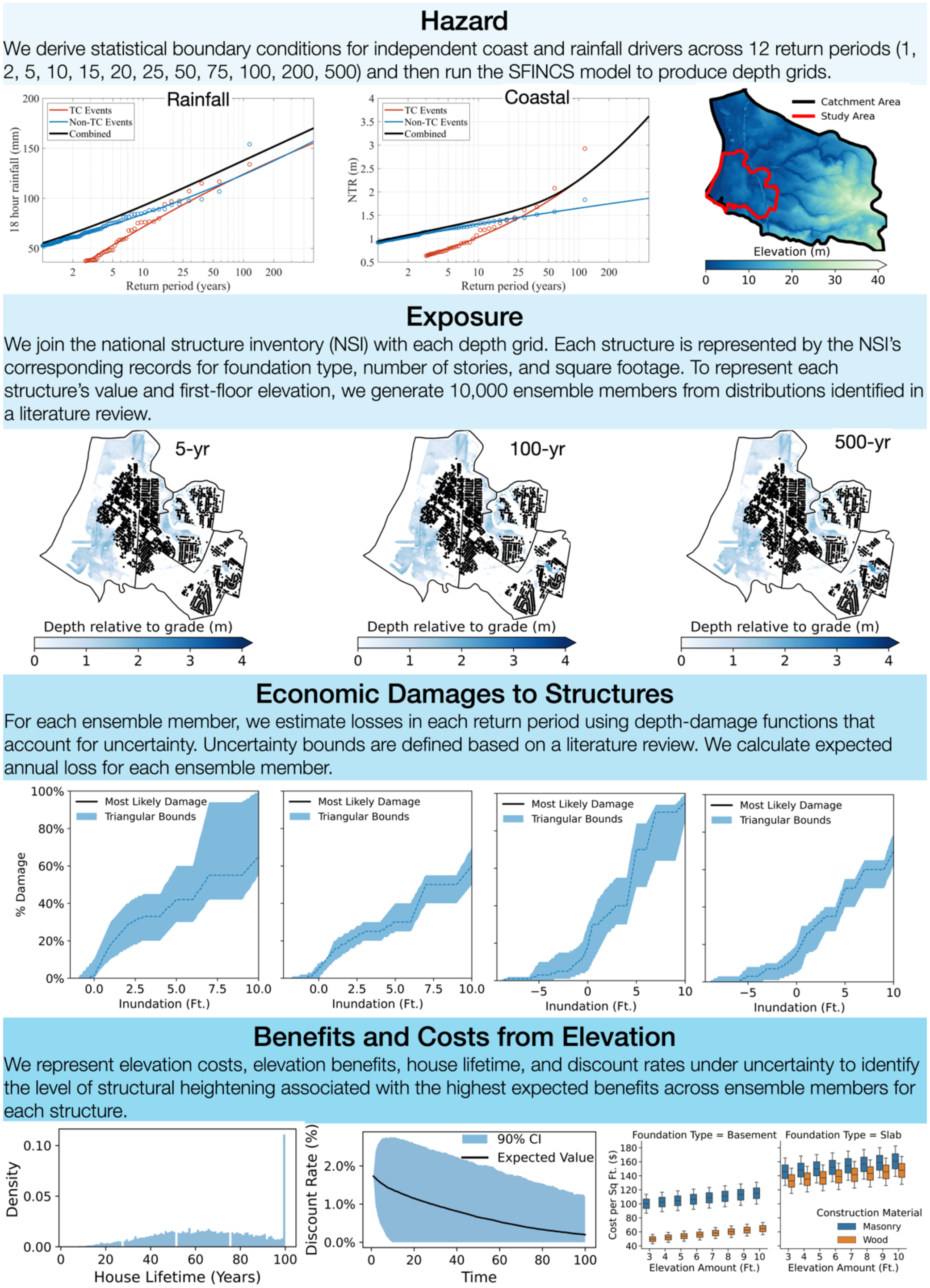

Figure S12. Quantitative workflow for representing uncertain inputs and system relationships. *The high-level steps for the hazard analysis, exposure representation, economic damage estimation, and calculation of benefits and costs for optimal elevation height are represented.. Small inset figures show data that represents a key input or output of an analysis step. Each of these steps are described in detail in the Methods section.*

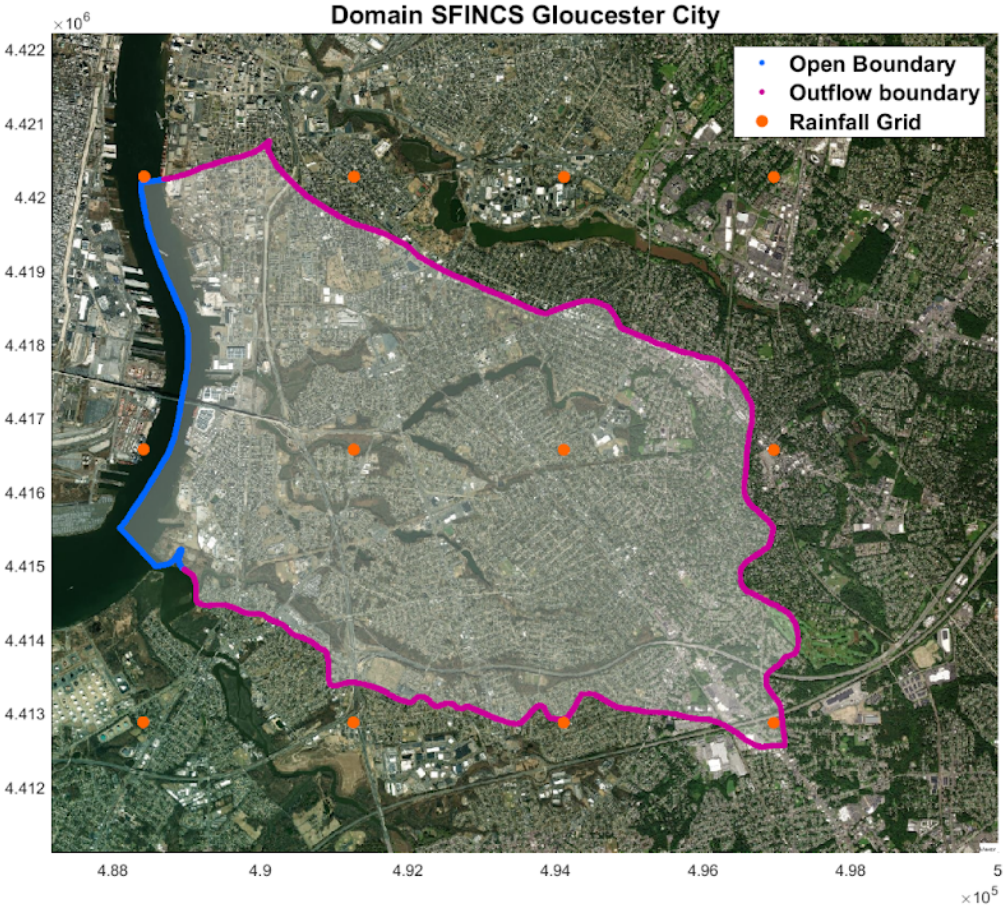

Figure S13. Domain used for the SFINCS flood model. *The domain covers the catchments of Little Timber Creek and Newton Creek, which surround Gloucester City. The blue boundary shows the open boundary where the water level scenarios were provided as boundary conditions for the simulations of coastal flooding. The purple boundary shows the inland boundaries of the domain, which were set as outflow boundaries. The orange points show the grid nodes of the RF data used as forcing for the simulations of pluvial flooding.*

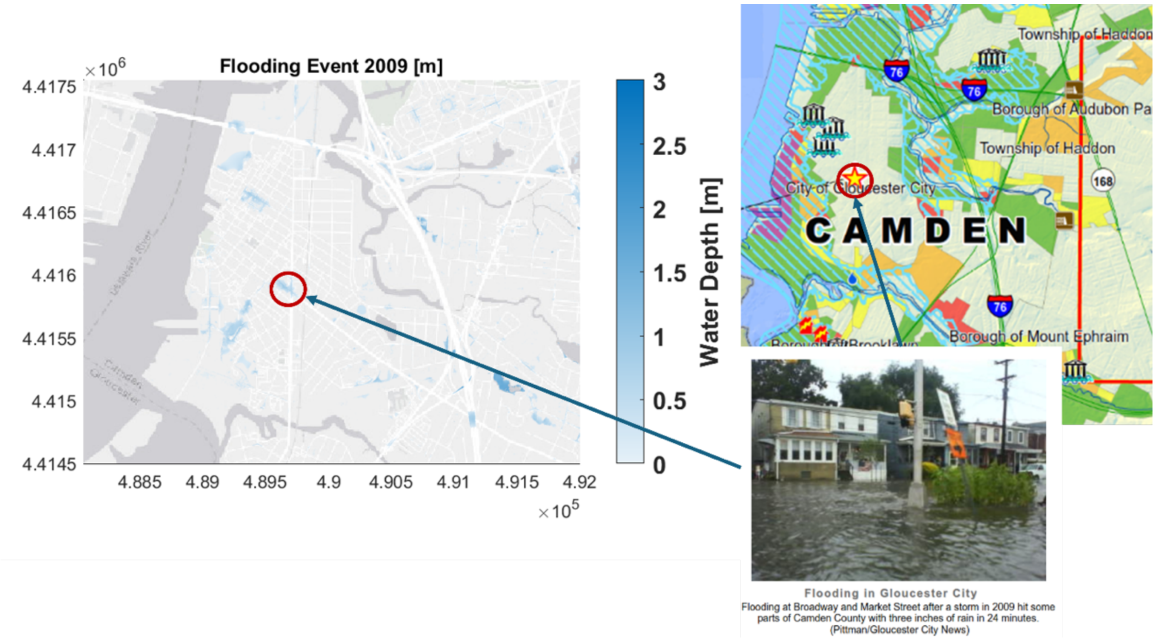

Figure S14. Flood map (left) resulting from the simulation (neglecting infiltration) of the 2009 flood event reported in the FEMA Risk Map (right). *Average water depths of 36cm are estimated for the intersection shown in the photo (red circle on left and right panels). Map on right taken from https://map1.msc.fema.gov/data/FRP/FRM_Coastal_34007_20170424.pdf?LOC=b0a94da948692dc0ccd7b08dc0c78418.*

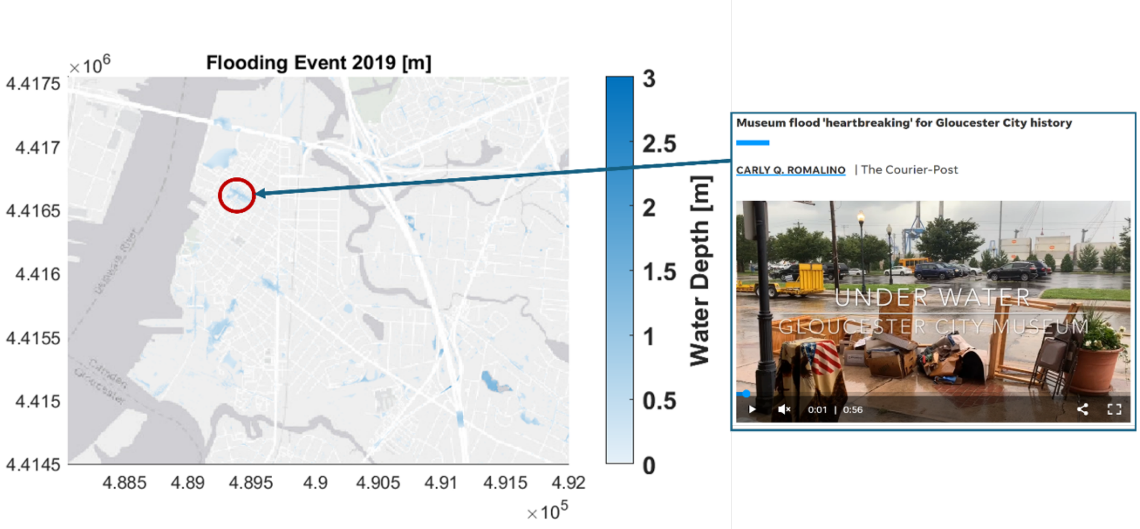

Figure S15. Comparing simulated flooding against known flooded location from 2019 event. *A news article (right) reported that the Historical Society Museum of Gloucester City experienced 3 feet of water in the basement. Under meteorological forcings of the flood event, the SFINCS model resulted in an average water depth of 68 cm for the location where the museum is located (red circle on left). News article was obtained from https://www.courierpostonline.com/story/news/local/south-jersey/2019/06/20/floodwaters-ruin-artifacts-gloucester-city-historical-society/1514980001/.*

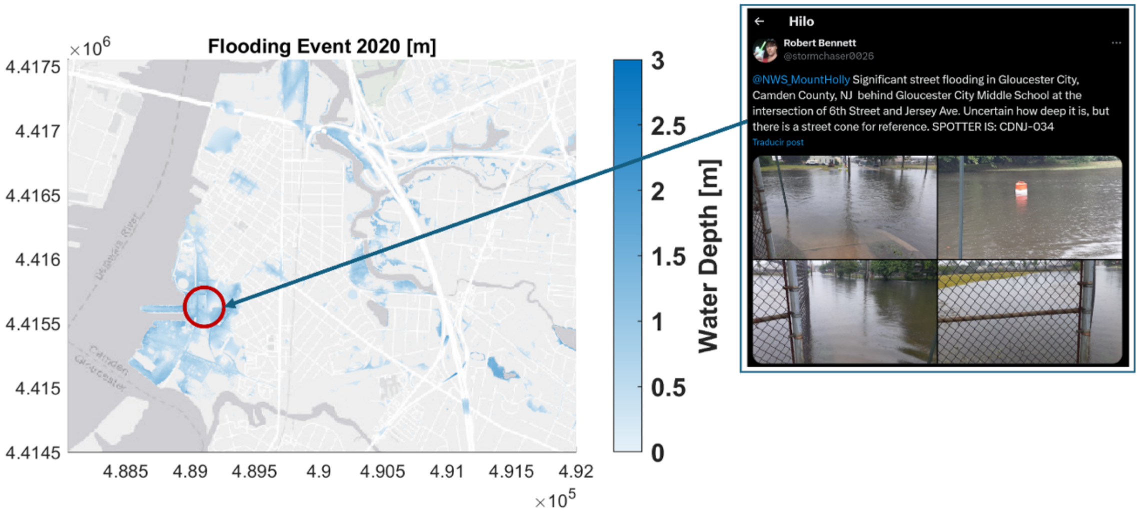

Figure S16. Comparing simulated flooding against known flooded location from 2020 event. *A tweet (right) reported flooded intersections by the Gloucester City Middle School. Under meteorological forcings of the flood event, the SFINCS model resulted in an average water depth of 72 cm in the area (red circle). Tweet was obtained from https://twitter.com/stormchaser0026/status/1281677885124771840.*

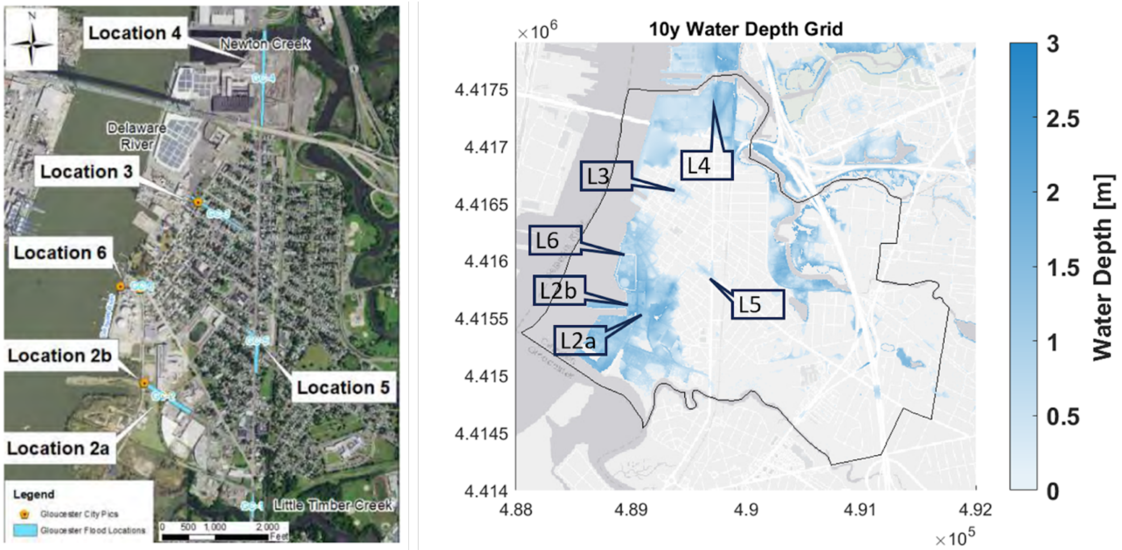

Figure S17: Comparing frequent design event flood locations known to flood frequently. *Left panel shows the locations in Gloucester City where the Camden County Municipal Utilities Authority (CCMUA) reports frequent flooding (source: Regional Flooding Study. Phase 1 Final Report. CDM Smith, 2023). Right panel shows the water depths from the simulation of the 10-year event.*

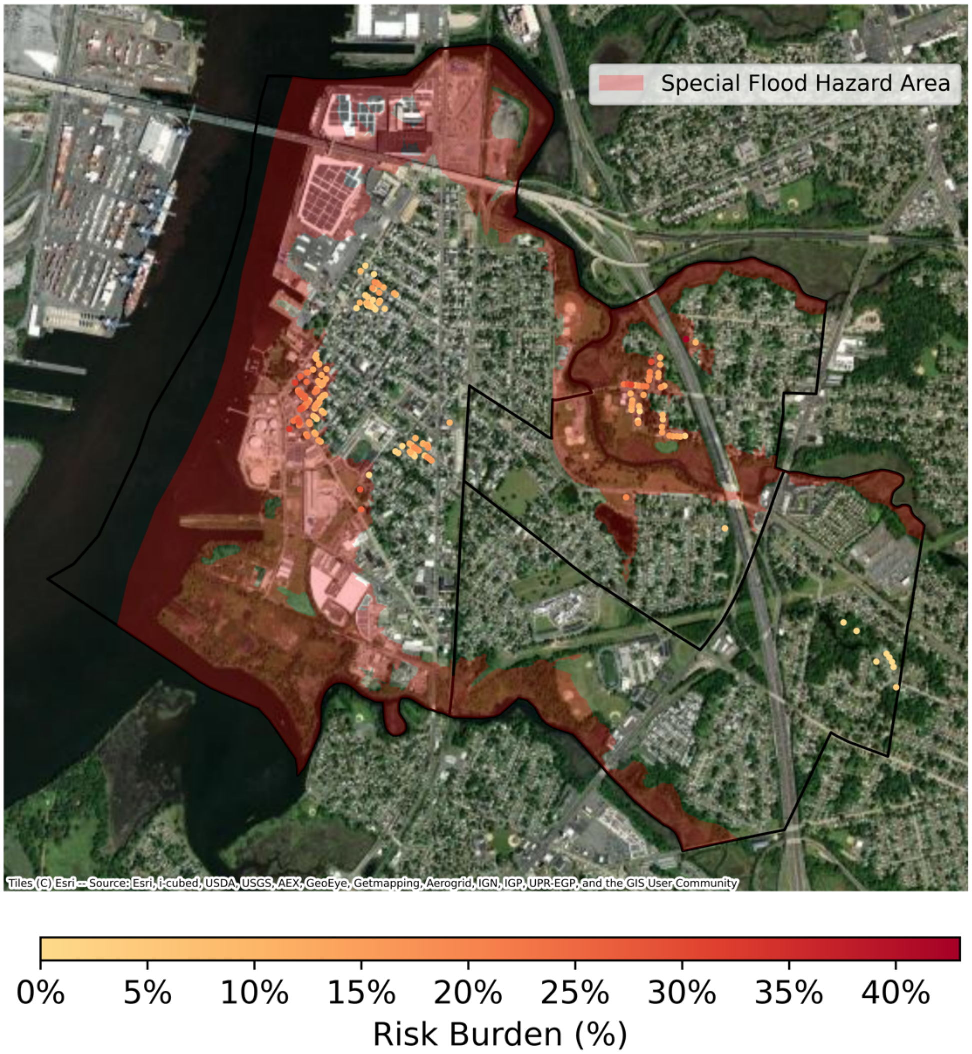

Figure S18: Comparing modeled flood-risk burden to special flood hazard area (SFHA) boundaries. *The SFHA denotes the 100-yr floodplain based on coastal and fluvial drivers. Properties that face modeled flood risk are mostly in the SFHA besides two clusters of properties that correspond to pluvial flooding locations from the 2019 and 2020 events.*

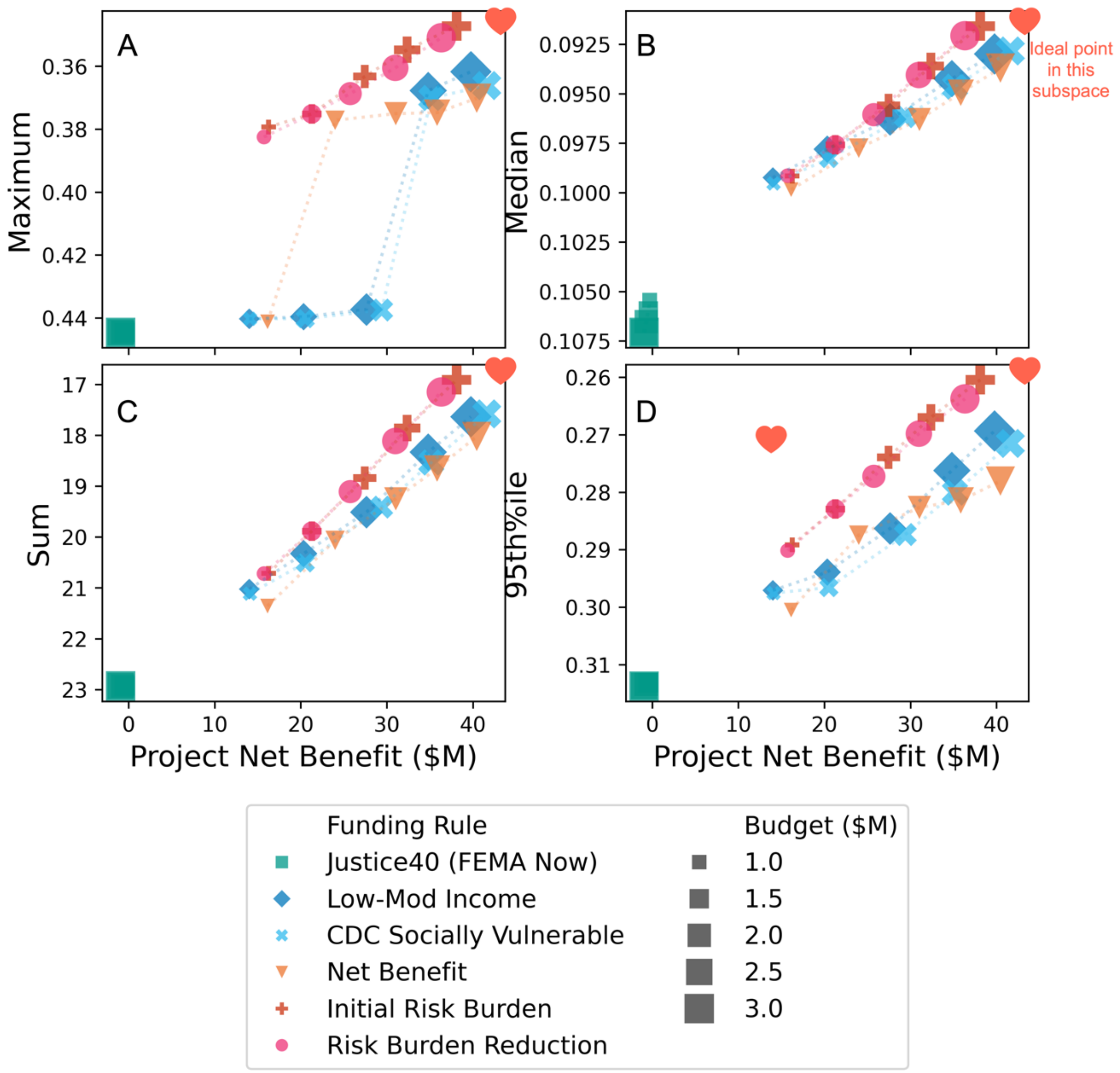

Figure S19: Performance of the considered policies for different ways of representing the distributive principle that lower risk burden is better. *Each panel shows the objective values different funding rules achieve as a function of the overall net benefit. The panels show different ways of representing the distributive principle that lower risk burden is better: maximum, consistent with the main manuscript (panel A), median (panel B), sum (panel C), and 95th percentile (panel D). The axes are ordered such that the ideal point in each panel is in the upper right corner. Note that objectives are only evaluated at each displayed point, but dashed lines are shown to enhance visual comparability. Note that there is only one Justice40 point because under the considered budgets, there is only one allocation that ensures the majority of benefits accrue in the Justice40 community.*

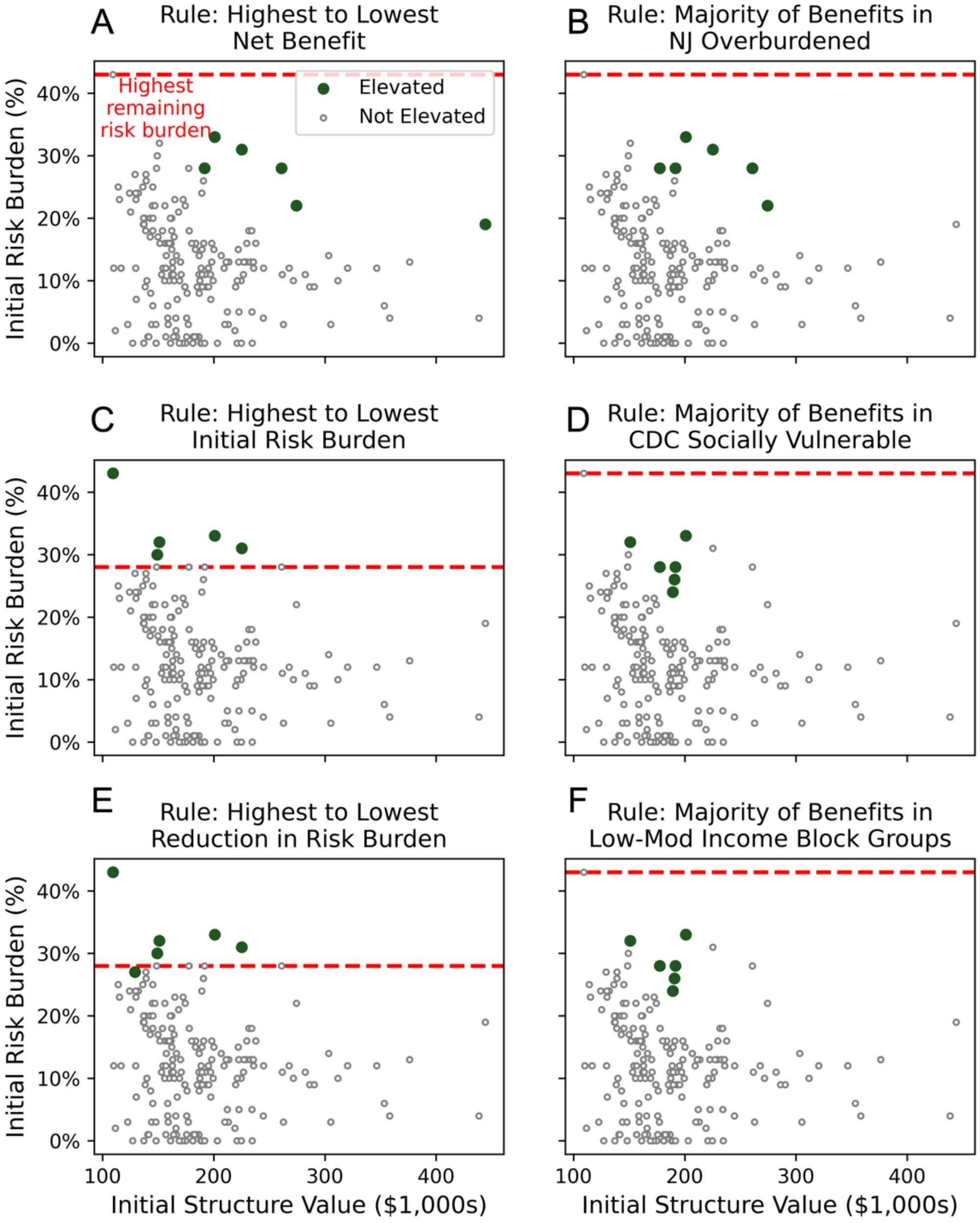

Figure S20.The distributional implications of considered funding rules under a $1M allocation budget, including the New Jersey overburdened communities definition. *Points represent individual structures in terms of their structure value and flood risk as a percentage of that value. Each panel shows the results of implementing a funding rule for a $1M budget (roughly the 75th percentile of all FEMA Hazard Mitigation Assistance flood elevation grants in 2022 dollars). Panels A, C, E (B, D, E) show household (community) rules. Houses receiving funding under each rule are plotted in green. Houses shown in gray do not receive funding. The dashed red line indicates the highest remaining risk burden after elevating houses, a quantity that indicates a more equitable investment when it is lower on the y-axis.*

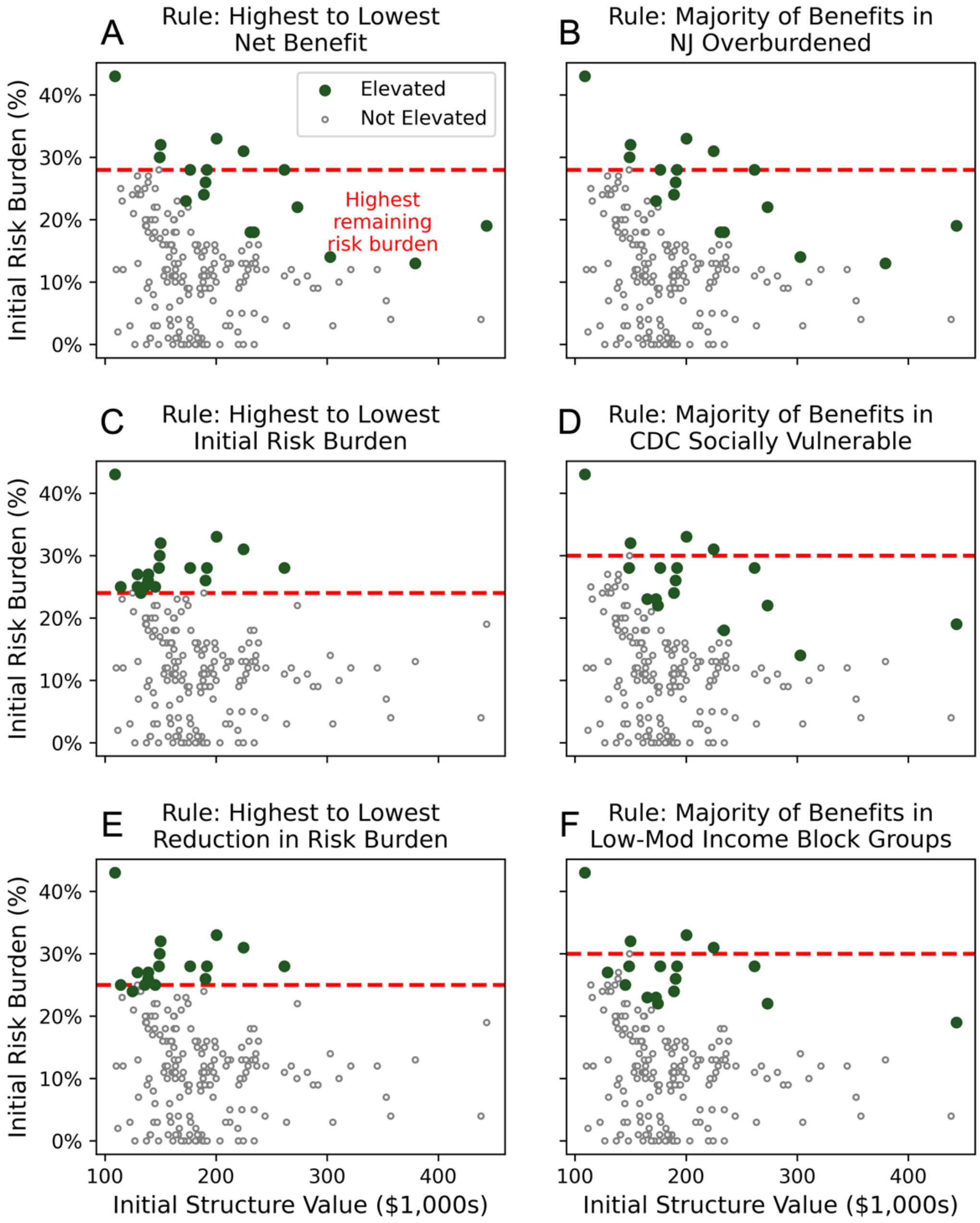

Figure S21: The distributional implications of considered funding rules under a $2.5M allocation budget, including the New Jersey overburdened communities definition. *Points represent individual structures in terms of their structure value and flood risk as a percentage of that value. Each panel shows the results of implementing a funding rule for a $2.5M budget. Panels A, C, E (B, D, E) show household (community) rules. Houses receiving funding under each rule are plotted in green. Houses shown in gray do not receive funding. The dashed red line indicates the highest remaining risk burden after elevating houses, a quantity that indicates a more equitable investment when it is lower on the y-axis.*

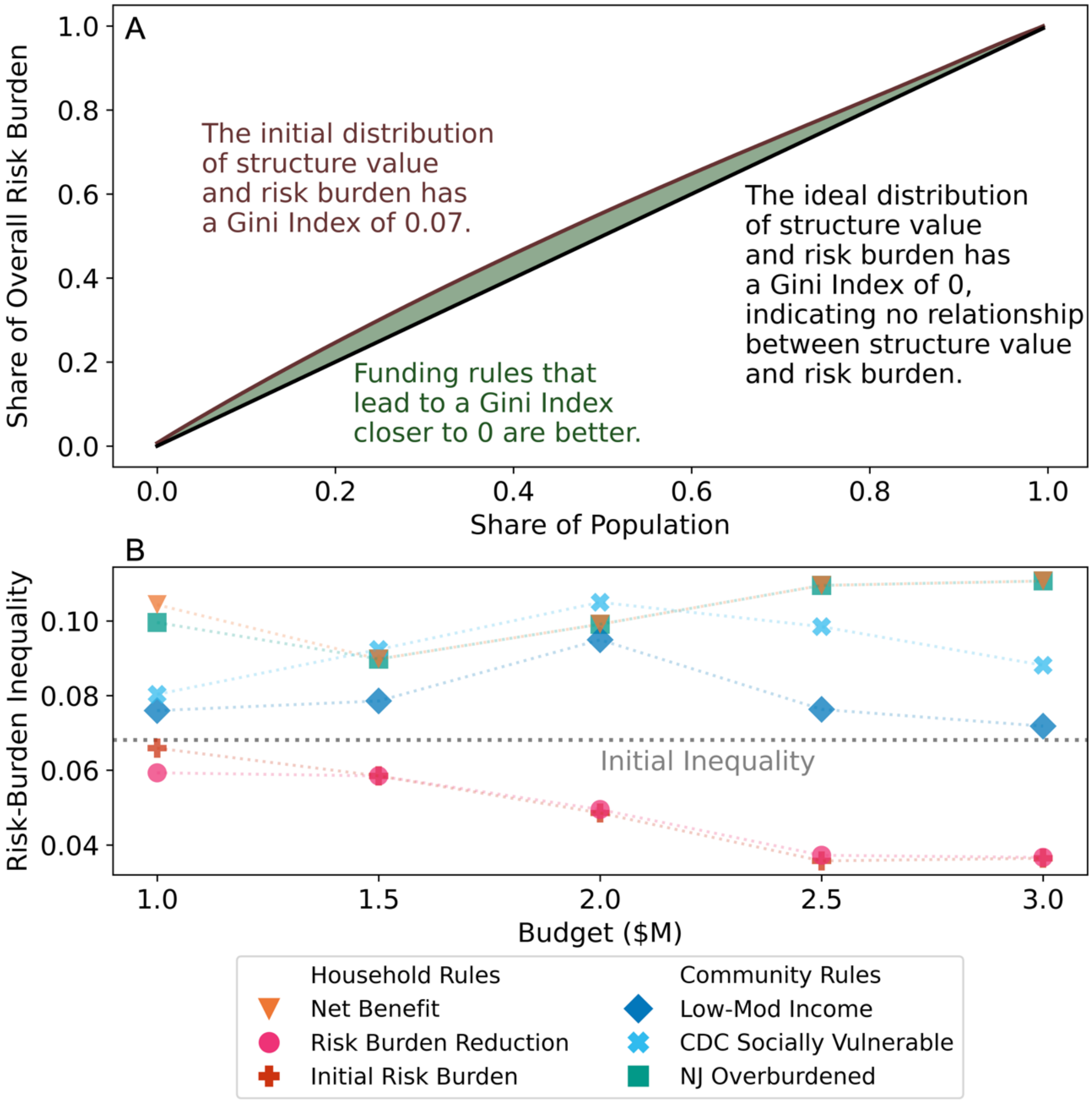

Figure S22: The cost of reducing risk-burden inequality, including the New Jersey overburdened communities definition. *Panel A shows the initial distribution of risk-burden inequality for properties sorted by structure value. Panel B shows the risk-burden inequality obtained by different funding rules under a variety of project budgets. A gray dashed line shows the initial inequality to contextualize which rules worsen or lessen inequality for different project costs. Note that objectives are only evaluated at each displayed point, but dashed lines are shown to enhance visual comparability.*

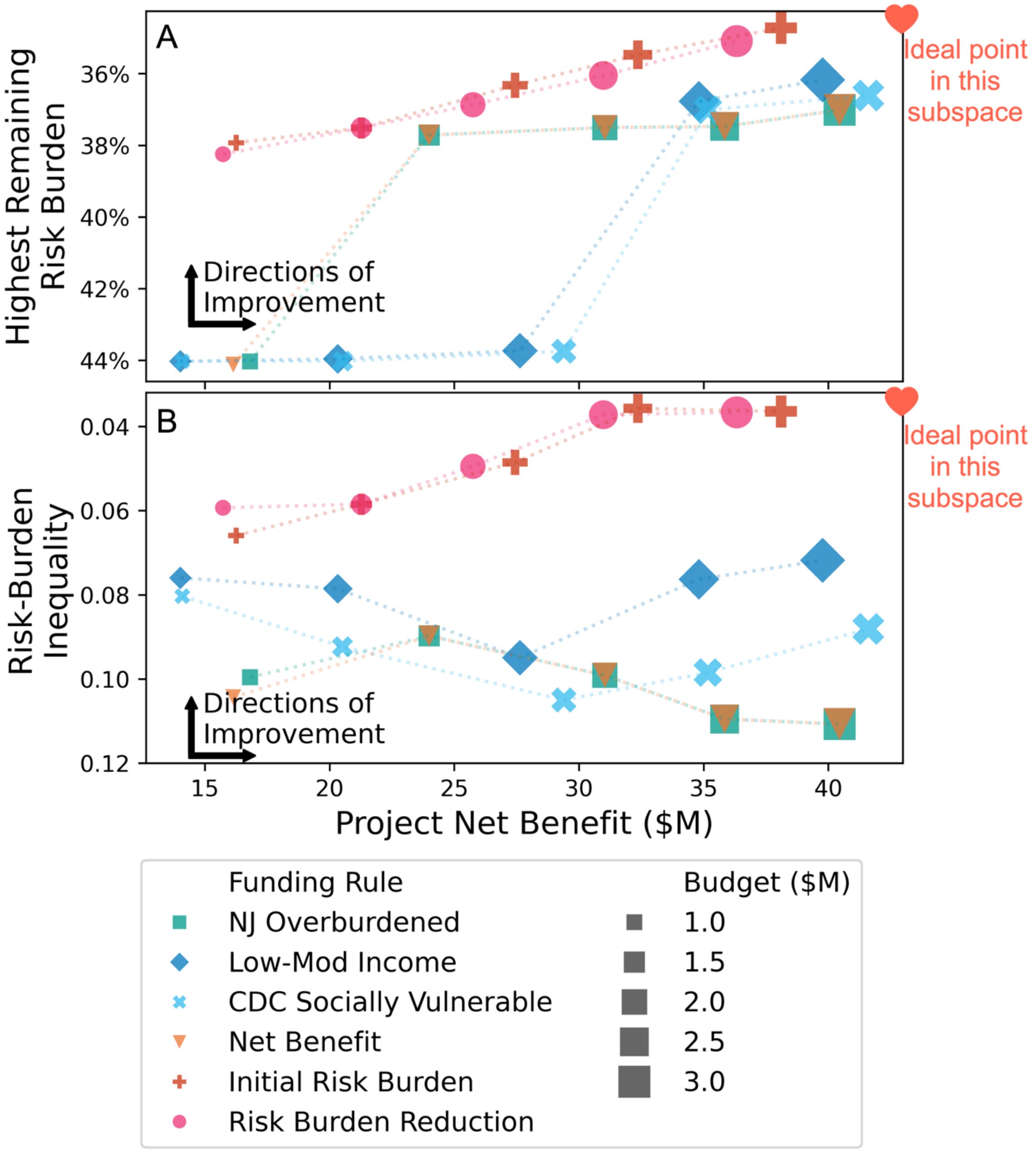

Figure S23: Performance of the funding rules across equity and efficiency objectives, including the New Jersey overburdened communities definition. *Panel A (B) shows the performance of funding rules in maximizing project net benefit and minimizing the highest remaining risk burden (risk-burden inequality). The axes are ordered such that the ideal point in each panel is in the upper right corner. Note that objectives are only evaluated at each displayed point, but dashed lines are shown to enhance visual comparability.*

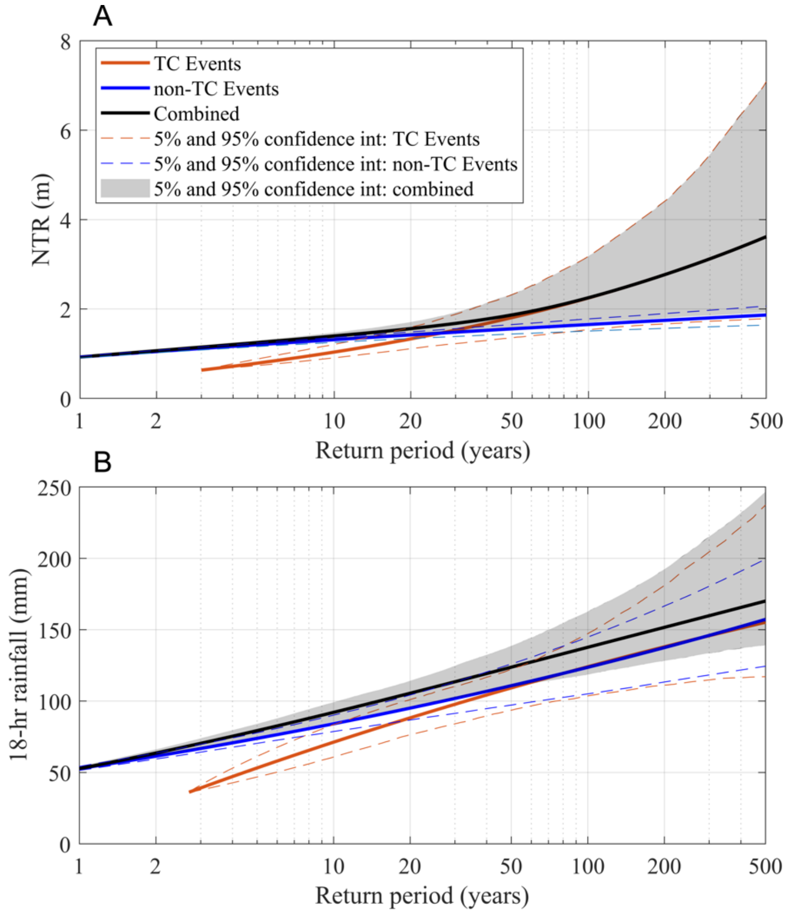

Figure S24: Bootstrapped confidence intervals for non-tidal residual and rainfall.

References

1. M. Zarekarizi, V. Srikrishnan, K. Keller, Neglecting uncertainties biases house-elevation decisions to manage riverine flood risks. *Nat. Commun.* **11**, 5361 (2020).

2. J. Doss-Gollin, K. Keller, A subjective Bayesian framework for synthesizing deep uncertainties in climate risk management. *Earths Future* **11** (2023).

3. A. Ciullo, J. H. Kwakkel, K. M. De Bruijn, N. Doorn, F. Klijn, Efficient or Fair? Operationalizing Ethical Principles in Flood Risk Management: A Case Study on the Dutch-German Rhine. *Risk Anal.* **40**, 1844–1862 (2020).

4. L. T. de Ruig, *et al.*, How the USA can benefit from risk-based premiums combined with flood protection. *Nat. Clim. Chang.* 1–4 (2022). https://doi.org/10.1038/s41558-022-01501-7.

5. F. Dottori, L. Mentaschi, A. Bianchi, L. Alfieri, L. Feyen, Cost-effective adaptation strategies to rising river flood risk in Europe. *Nat. Clim. Chang.* 1–7 (2023). https://doi.org/10.1038/s41558-022-01540-0.

6. E. Tate, A. Strong, T. Kraus, H. Xiong, Flood recovery and property acquisition in Cedar Rapids, Iowa. *Nat. Hazards* **80**, 2055–2079 (2016).

7. J. C. J. H. Aerts, *et al.*, Climate adaptation. Evaluating flood resilience strategies for coastal megacities. *Science* **344**, 473–475 (2014).

8. L. T. de Ruig, T. Haer, H. de Moel, W. J. W. Botzen, J. C. J. H. Aerts, A micro-scale cost-benefit analysis of building-level flood risk adaptation measures in Los Angeles. *Water Resources and Economics* **32**, 100147 (2020).

9. T. Leijnse, M. van Ormondt, K. Nederhoff, A. van Dongeren, Modeling compound flooding in coastal systems using a computationally efficient reduced-physics solver: Including fluvial, pluvial, tidal, wind- and wave-driven processes. *Coast. Eng.* **163**, 103796 (2021).

10. B. F. Sanders, *et al.*, Large and inequitable flood risks in Los Angeles, California. *Nature Sustainability* 1–11 (2022). https://doi.org/10.1038/s41893-022-00977-7.

11. B. Merz, H. Kreibich, R. Schwarze, A. Thieken, Review article "Assessment of economic flood damage." *Nat. Hazards Earth Syst. Sci.* **10**, 1697–1724 (2010).

12. H. de Moel, L. M. Bouwer, J. C. J. H. Aerts, Uncertainty and sensitivity of flood risk calculations for a dike ring in the south of the Netherlands. *Sci. Total Environ.* **473–474**, 224–234 (2014).

13. P. Maduwantha, *et al.*, A multivariate statistical framework for mixed populations in compound flood analysis. *EGUsphere* 1–27 (2024). https://doi.org/10.5194/egusphere-2024-1122.

14. D. H. Kitzmiller, W. Wu, Z. Zhang, N. Patrick, X. Tan, The Analysis of Record for Calibration: A High-Resolution Precipitation and Surface Weather Dataset for the United States in (2018), pp. H41H-06.

15. P. S. Smitha, B. Narasimhan, K. P. Sudheer, H. Annamalai, An improved bias correction method of daily rainfall data using a sliding window technique for climate change impact assessment. *J. Hydrol.* **556**, 100–118 (2018).

16. R. Jane, L. Cadavid, J. Obeysekera, T. Wahl, Multivariate statistical modelling of the drivers of compound flood events in south Florida. *Nat. Hazards Earth Syst. Sci.* **20**, 2681–2699 (2020).

17. P. Camus, *et al.*, Regional analysis of multivariate compound coastal flooding potential around Europe and environs: sensitivity analysis and spatial patterns. *Nat. Hazards Earth Syst. Sci.* **21**, 2021–2040 (2021).

18. C. W. Landsea, J. L. Franklin, Atlantic Hurricane Database Uncertainty and Presentation of a New Database Format. *Mon. Weather Rev.* **141**, 3576–3592 (2013).

19. New Jersey Water Science Center, Summary of Flooding in New Jersey Caused by Hurricane Irene, August 27–30, 2011. (2011). Available at: https://www.usgs.gov/news/summary-flooding-new-jersey-caused-hurricane-irene-august-27-30-2011 [Accessed 29 April 2024].

20. H. Kim, *et al.*, On the generation of high-resolution probabilistic design events capturing the joint occurrence of rainfall and storm surge in coastal basins. *Int. J. Climatol.* (2022). https://doi.org/10.1002/joc.7825.

21. J. J. Danielson, *et al.*, Topobathymetric Elevation Model Development using a New Methodology: Coastal National Elevation Database. *coas* **76**, 75–89 (2016).

22. B. R. Röbke, *et al.*, Rapid Assessment of Tsunami Offshore Propagation and Inundation with D-FLOW Flexible Mesh and SFINCS for the 2011 Tōhoku Tsunami in Japan. *J. Mar. Sci. Eng.* **9**, 453 (2021).

23. United States Army Corps of Engineers, Creating Land Cover, Manning's n values, and % Impervious Layers. (2024). Available at: https://www.hec.usace.army.mil/confluence/rasdocs/r2dum/latest/developing-a-terrain-model-and-geospatial-layers/creating-land-cover-mannings-n-values-and-impervious-layers [Accessed 22 March 2024].

24. D. Molinari, K. M. De Bruijn, J. T. Castillo-Rodríguez, G. T. Aronica, L. M. Bouwer, Validation of flood risk models: Current practice and possible improvements. *International Journal of Disaster Risk Reduction* **33**, 441–448 (2019).

25. P. Bates, Fundamental limits to flood inundation modelling. *Nature Water* **1**, 566–567 (2023).

26. M. Hino, E. Nance, Five ways to ensure flood-risk research helps the most vulnerable. *Nature* **595**, 27–29 (2021).

27. B. Tellman, *et al.*, Satellite imaging reveals increased proportion of population exposed to floods. *Nature* **596**, 80–86 (2021).

28. Risk Mapping, Assessment and Planning (Risk MAP). Available at: https://www.fema.gov/flood-maps/tools-resources/risk-map [Accessed 28 August 2024].

29. Flood Risk Map: Camden County Coastal Project Area, New Jersey. (2016).

30. O. E. J. Wing, *et al.*, Simulating historical flood events at the continental scale: observational validation of a large-scale hydrodynamic model. *Nat. Hazards Earth Syst. Sci.* **21**, 559–575 (2021).

31. P. M. Orton, *et al.*, A validated tropical-extratropical flood hazard assessment for New York Harbor. *Journal of Geophysical Research: Oceans* **121**, 8904–8929 (2016).

32. Camden County, New Jersey, *draft Camden County Hazard Mitigation Plan. Section 9.15: City of Gloucester.* [Preprint] (2021). Available at: https://www.camdencounty.com/wp-content/uploads/2021/02/Draft-9.15-City-of-Gloucester_082521san.pdf.

33. Flood Event Viewer. Available at: https://stn.wim.usgs.gov/FEV/ [Accessed 28 August 2024].

34. MyCoast: New Jersey - MyCoast. (2019). Available at: https://mycoast.org/nj [Accessed 28 August 2024].

35. C. Kousky, H. Kunreuther, Addressing Affordability in the National Flood Insurance Program. *J. of Extr. Even.* **01**, 1450001 (2014).

36. B. Wilson, E. Tate, C. T. Emrich, Flood recovery outcomes and disaster assistance barriers for vulnerable populations. *Front. Water* **3**, 752307 (2021).

37. Federal Emergency Management Agency, Hazus 6.0 Inventory Technical Documentation. (2022). Available at: https://www.fema.gov/sites/default/files/documents/fema_hazus-6-inventory-technical-manual.pdf [Accessed 5 March 2024].

38. Federal Emergency Management Agency, Hazus 5.1 Flood Technical Manual. (2022). Available at: https://www.fema.gov/sites/default/files/documents/fema_hazus-flood-model-technical-manual-5-1.pdf [Accessed 5 March 2024].

39. United States Army Corps of Engineers, National Structure Inventory Technical Documentation. (2022). Available at: https://www.hec.usace.army.mil/confluence/nsi/technicalreferences/latest/technical-documentation [Accessed 5 March 2024].

40. Shultz Steven, Correcting HAZUS General Building Stock Structural Replacement Cost Data for Single-Family Residences. *Nat. Hazards Rev.* **18**, 04017015 (2017).

41. Shultz Steven, Accuracy of HAZUS General Building Stock Data. *Nat. Hazards Rev.* **18**, 04017012 (2017).

42. R. B. Mostafiz, *et al.*, Comparison of Neighborhood-Scale, Residential Property Flood-Loss Assessment Methodologies. *Front. Environ. Sci. Eng. China* **9** (2021).

43. N. Saint-Geours, F. Grelot, J.-S. Bailly, C. Lavergne, Ranking sources of uncertainty in flood damage modelling: a case study on the cost-benefit analysis of a flood mitigation project in the Orb Delta, France. *J. Flood Risk Manag.* **8**, 161–176 (2015).

44. A. B. Pollack, I. Sue Wing, C. Nolte, Aggregation bias and its drivers in large-scale flood loss estimation: A Massachusetts case study. *Journal of Flood Risk* (2022).

45. J. D. Gourevitch, *et al.*, Unpriced climate risk and the potential consequences of overvaluation in US housing markets. *Nat. Clim. Chang.* 1–8 (2023). https://doi.org/10.1038/s41558-023-01594-8.

46. C. Kousky, M. Walls, Floodplain conservation as a flood mitigation strategy: Examining costs and benefits. *Ecol. Econ.* **104**, 119–128 (2014).

47. M. Montgomery, H. Kunreuther, Pricing Storm Surge Risks in Florida: Implications for Determining Flood Insurance Premiums and Evaluating Mitigation Measures. *Risk Anal.* **38**, 2275–2299 (2018).

48. I. A. Bick, *et al.*, Rising seas, rising inequity? Communities at risk in the San Francisco bay area and implications for adaptation policy. *Earths Future* **9** (2021).

49. O. E. J. Wing, *et al.*, Inequitable patterns of US flood risk in the Anthropocene. *Nat. Clim. Chang.* **12**, 156–162 (2022).

50. I. Hosseini-Shakib, A. Alipour, B. S. Lee, V. Srikrishnan, S. Sharma, What drives uncertainty surrounding riverine flood risks? *J. Hydrol.* 131055 (2024). https://doi.org/10.1016/j.jhydrol.2024.131055.

51. E. Tate, C. Muñoz, J. Suchan, Uncertainty and sensitivity analysis of the HAZUS-MH flood model. *Nat. Hazards Rev.* **16**, 04014030 (2015).

52. H. de Moel, J. C. J. H. Aerts, Effect of uncertainty in land use, damage models and inundation depth on flood damage estimates. *Nat. Hazards* **58**, 407–425 (2011).

53. A. Krause, A. Martin, M. Fix, Uncertainty in automated valuation models: Error-based versus model-based approaches. *Journal of Property Research* **37**, 308–339 (2020).

54. C. Nolte, High-resolution land value maps reveal underestimation of conservation costs in the United States. *Proc. Natl. Acad. Sci. U. S. A.* **117**, 29577–29583 (2020).

55. A. D. Nowak, P. S. Smith, Quality-Adjusted House Price Indexes. *American Economic Review: Insights* **2**, 339–356 (2020).

56. C. Nolte, *et al.*, Data Practices for Studying the Impacts of Environmental Amenities and Hazards with Nationwide Property Data. *Land Econ.* (2023). https://doi.org/10.3368/le.100.1.102122-0090R.

57. United States Army Corps of Engineers, North Atlantic Coast Comprehensive Study: Resilient Adaptation to Increasing Risk, Physical Depth Damage Function Summary Report. (2015). Available at: https://www.nad.usace.army.mil/Portals/40/docs/NACCS/10A_PhysicalDepthDmgFxSummary_26Jan2015.pdf [Accessed 5 March 2024].

58. C. R. Harris, *et al.*, Array programming with NumPy. *Nature* **585**, 357–362 (2020).

59. Creators The pandas development team, *pandas-dev/pandas: Pandas*.

60. Creators The geopandas development team, *geopandas/geopandas: v0.13.2*.

61. S. Gillies, Others, *Rasterio: geospatial raster I/O for Python programmers* (Mapbox, 2013).

62. V. Srikrishnan, *random-discount: Repository for uncertain discounting model calibration and prediction* (Github, 2023).

63. R. G. Newell, W. A. Pizer, Discounting the distant future: how much do uncertain rates increase valuations? *J. Environ. Econ. Manage.* **46**, 52–71 (2003).

64. R. G. Newell, W. A. Pizer, B. C. Prest, A Discounting Rule for the Social Cost of Carbon. *Journal of the Association of Environmental and Resource Economists* **9**, 1017–1046 (2022).

65. M. D. Bauer, G. D. Rudebusch, The rising cost of climate change: Evidence from the bond market. *Rev. Econ. Stat.* 1–45 (2021). https://doi.org/10.1162/rest_a_01109.

66. K. Keller, C. Helgeson, V. Srikrishnan, Climate Risk Management. *Annual Review of Earth and Planetary Sciences* **49**, 95–116 (2021).

67. H. Wiley, C. Kousky, Speeding up post-disaster housing buyouts. (2021). Available at: https://www.preventionweb.net/publication/speeding-post-disaster-housing-buyouts [Accessed 6 March 2024].

68. J. C. J. H. Aerts, A Review of Cost Estimates for Flood Adaptation. *Water* **10**, 1646 (2018).

69. J. C. J. H. Aerts, W. J. W. Botzen, H. de Moel, M. Bowman, Cost estimates for flood resilience and protection strategies in New York City. *Ann. N. Y. Acad. Sci.* **1294**, 1–104 (2013).

70. FEMA, Homeowner’s Guide to Retrofitting Second Edition (FEMA P-312). (2009). Available at: https://www.austintexas.gov/sites/default/files/files/Watershed/flood/fema_p312_a.pdf [Accessed 6 March 2024].

71. FEMA, Third Edition of FEMA P-312, Homeowner’s Guide to Retrofitting,. (2014). Available at: https://www.fema.gov/sites/default/files/2020-08/FEMA_P-312.pdf [Accessed 6 March 2024].

72. National Institute of Building Sciences, Natural Hazard Mitigation Saves: 2019 Report. (2019). Available at: https://www.nibs.org/projects/natural-hazard-mitigation-saves-2019-report [Accessed 6 March 2024].

73. B. McMann, M. Schulze, H. Sprague, K. Smyth, 2017 Louisiana Coastal Master Plan-Appendix A: Project Definition. (2017). Available at: https://coastal.la.gov/wp-content/uploads/2017/04/Appendix-A_FINAL_04.04.2017.pdf [Accessed 6 March 2024].

74. U.S. Bureau of Labor Statistics, Employment Cost Index: Wages and salaries for Private industry workers in Construction. Deposited 19 December 2023.

75. US Census Bureau, Price Indexes for New Single-Family Houses Under Construction. Deposited 19 December 2023.

76. The Department of Homeland Security, The Department of Homeland Security (DHS) Notice of Funding Opportunity (NOFO) Fiscal Year 2022 Flood Mitigation Assistance. [Preprint] (2022). Available at: https://www.fema.gov/sites/default/files/documents/fema_fy22-fma-nofo_08052022_0.pdf [Accessed 19 October 2022].

77. The Department of Homeland Security, The Department of Homeland Security (DHS) Notice of Funding Opportunity (NOFO) Fiscal Year 2022 Building Resilient Infrastructure and Communities. [Preprint] (2022). Available at: https://www.fema.gov/sites/default/files/documents/fema_fy22-bric-nofo_08052022.pdf [Accessed 19 October 2022].

78. K. Lowe, S. Reckhow, J. F. Gainsborough, Capacity and equity: Federal funding competition between and within metropolitan regions. *J. Urban Aff.* **38**, 25–41 (2016).

79. B. Q. Huynh, *et al.*, Mitigating allocative tradeoffs and harms in an environmental justice data tool. *Nature Machine Intelligence* **6**, 187–194 (2024).

80. D. Pisinger, Where are the hard knapsack problems? *Comput. Oper. Res.* **32**, 2271–2284 (2005).

81. FEMA, Flood Mitigation Assistance Fiscal Year 2021 Subapplication Status. (2022). Available at: https://www.fema.gov/grants/mitigation/floods/flood-mitigation-assistance-fy-2021-subapplication-status [Accessed 19 October 2022].

82. FEMA, Building Resilient Infrastructure and Communities FY 2021 Subapplication and Selection Status. (2022). Available at: https://www.fema.gov/grants/mitigation/building-resilient-infrastructure-communities/after-apply/fy-2021-subapplication-status [Accessed 19 October 2022].

83. Executive Office of the President, Tackling the Climate Crisis at Home and Abroad. *Fed. Regist.* [Preprint] (2021). Available at: https://www.federalregister.gov/documents/2021/02/01/2021-02177/tackling-the-climate-crisis-at-home-and-abroad.

84. Sanders Brett F., *et al.*, Quantifying Social Inequalities in Flood Risk. *ASCE OPEN: Multidisciplinary Journal of Civil Engineering* **2**, 04024004 (2024).

85. A. B. Pollack, C. Helgeson, C. Kousky, K. Keller, Developing more useful equity measurements for flood-risk management. *Nature Sustainability* 1–10 (2024). https://doi.org/10.1038/s41893-024-01345-3.

86. New Jersey Department of Environmental Protection, What are Overburdened Communities (OBC)? *Environmental Justice* (2022). Available at: https://dep.nj.gov/ej/communities/ [Accessed 16 February 2024].
